# Dielectric stabilization controls excited-state proton transfer and ion pair dynamics in organic solvents†

**DOI:** 10.1039/d5sc03404c

**Published:** 2025-06-30

**Authors:** Amar Raj, Pragya Verma, Andrei Beliaev, Pasi Myllyperkiö, Tatu Kumpulainen

**Affiliations:** a Department of Chemistry/Nanoscience Center, University of Jyväskylä P.O. Box 35 Jyväskylä FI-40014 Finland tatu.s.kumpulainen@jyu.fi; b Department of Physical Chemistry, University of Geneva 30 Quai Ernest Ansermet Geneva Switzerland

## Abstract

Excited-state proton transfer (ESPT) in aprotic organic solvents has received limited attention due to their inability to accept protons. However, bimolecular ESPT from a photoacid to an organic base in such media enables systematic studies on the influence of macroscopic solvent parameters on the ESPT as demonstrated in this work. The full photocycle starting from initial deprotonation in a hydrogen-bonded donor–acceptor complex to full dissociation in the excited state followed by slow recombination in the ground state was characterized by various spectroscopic methods in solvent mixtures of varying polarity. The initial deprotonation producing contact ion pairs is ultrafast (sub-100 fs) and requires minimal solvent reorganization. The contact ion pairs dissociate *via* a distinct intermediate, the so-called solvent-separated ion pair, preceding the fully dissociated free ion pairs. The time scale of the ion pair dynamics is dominated by viscosity whereas the yield is determined by the polarity. In low polarity solvents (*ε*_r_ < 10), the population is trapped as solvent-separated ion pairs and full dissociation becomes operative only at intermediate polarity. Ground-state recombination of the intermediate ion pair species is fast and thus a significant population of fully dissociated ground-state ions is produced only above intermediate polarities.

## Abstract 2

Excited-state proton transfer and ion pair dynamics is controlled by solvent polarity in organic solvents. Polar solvents facilitate full separation into free ion pairs while only solvent-separated ion pairs are formed in low polarity solvents.

## Graphical abstract

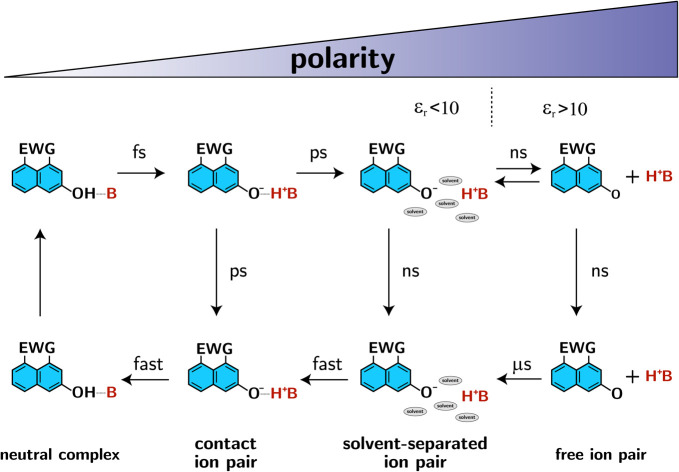

## 1 Introduction

Proton transfer is a ubiquitous phenomenon that finds widespread application in various chemical, biological, and technological processes.^1–8^ One prominent example of this reaction is intermolecular excited-state proton transfer (ESPT) that involves the transfer of a proton from an excited-state acid, also known as a photoacid, to an acceptor (a base or solvent) leading to the formation of an ion pair.^9–13^ The origin of the photoacidity is generally attributed to the charge-transfer character of the excited state of the photoacid and bears similarity to proton-coupled electron transfer.^14,15^ The charge transfer reduces the electron density at the proton donating group (typically an –OH group) resulting in dramatic increase of the acidity.^16^ The charge-transfer character can be increased by the introduction of strong electron-withdrawing groups on the aromatic skeleton and has been the main strategy for the development of even stronger photoacids with negative excited-state p*K*_a_ values 
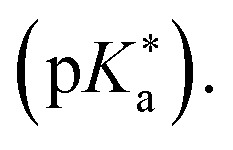
^17,18^

As the process is strongly influenced by the solvent, investigating the effect of various solvent parameters can provide valuable insight into the fundamental properties of proton transfer in solution, as has been demonstrated for electron-transfer reactions.^19–22^ The ESPT reaction exhibits many similarities to other photochemical reactions, where the chemical reactivity in solutions is often correlated with empirical solvent scales or macroscopic solvent parameters derived from a continuous description of the liquid.^23–25^ However, in ESPT to the solvent, the solvent acts as an active reaction partner thus complicating systematic studies on pure solvent effects.^26^ The ESPT rate of strong photoacids in protic solvents is often limited by the reorganization time of the solvation shell and the hydrogen-bond network of the accepting solvent.^16,27^ Furthermore, the number of solvents that can act as a proton acceptor is very limited even for the strongest photoacids.^17,18,28^

Focusing on bimolecular ESPT from a photoacid to an organic base can overcome the above challenges.^29–32^ However, elucidating the intricate interplay between different macroscopic solvent parameters is challenging since most solvent parameters vary from solvent to solvent. For example, variation in the dielectric constants may result in changes in other properties, such as proticity, viscosity or refractive index, which can lead to a confounding effect on the reaction of interest. One solution to circumvent this challenge is to use solvent mixtures, which has been demonstrated to be an effective approach for studying solvent effects on a broad range of photochemical reactions.^33–37^

**Scheme 1:**
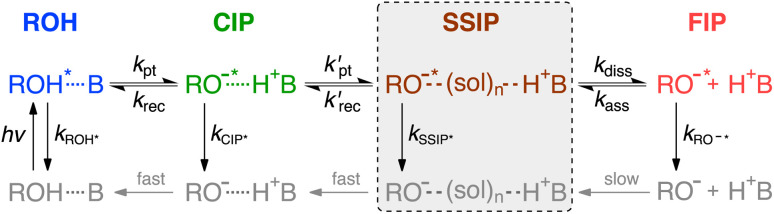
The Eigen–Weller model for intermolecular excited-state proton transfer from an acid (ROH) to a base (B). The first step involves a short-range proton transfer in the hydrogen-bonded complex (ROH, blue) yielding contact ion pairs (CIPs, green). Breakage of the direct hydrogen bond results in the formation of solvent-separated ion pairs (SSIPs, brown) that can further dissociate into free ion pairs (FIPs, red). The additional SSIP intermediate is highlighted inside the dashed box.

ESPT to solvent is commonly explained according to the two-step Eigen–Weller model illustrated in Scheme 1.^27,38^ The first step involves a short-range proton transfer in the encounter complex (ROH, blue in Scheme 1) resulting in the formation of a contact ion pair (CIP, green in Scheme 1) followed by a diffusion-controlled separation into a free ion pair (FIP, red in Scheme 1). In the case of bimolecular ESPT in polar aprotic organic solvents, the so-called solvent-separated ion pair (SSIP, brown in Scheme 1) has been identified as an additional reaction intermediate between the CIP and FIP.^29–31,39^ In SSIP, the ion pair is separated by a few solvent molecules but is still held together by the Coulomb interaction. Similar ion pair species, usually termed as tight and loose ion pairs, have also been identified in photoinduced electron transfer reactions but experimental investigation of these elusive intermediates is challenging.^40–42^ Population dynamics of the ion pairs directly impacts the yield of free ions that are usually the desired reaction product. Therefore, a thorough understanding of the solvent effects on the formation of ion pair species is of paramount importance for several processes, such as photoacid/base catalysis,^43^ photoredox catalysis^44,45^ and proton-coupled electron transfer.^46^

**Chart 1:**
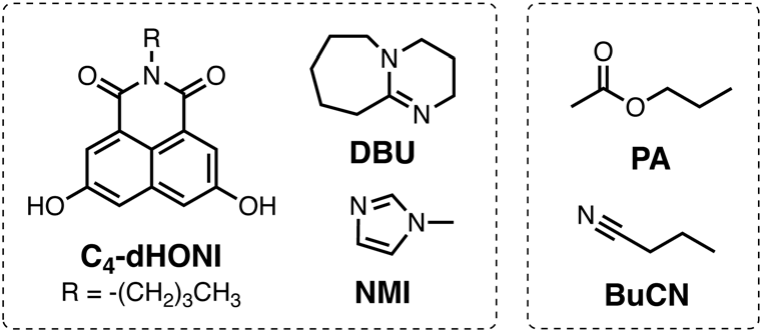
Chemical structures of the C_4_-dHONI photoacid, weak (NMI) and strong (DBU) organic bases and the solvents (PA and BuCN).

The objective of this study is to investigate the influence of dielectric stabilization on bimolecular ESPT and ion pair formation in organic media. To achieve this goal, we utilized a binary mixture of propyl acetate (PA) and butyronitrile (BuCN) as the solvent system and a 1,8-naphthalimide based photoacid (C_4_-dHONI) and a weak organic base (*N*-methylimidazole, NMI) as the reactant pair (Chart 1). The ground- and excited-state p*K*_a_ values of C_4_-dHONI are p*K*_a_ = 8.8 and 
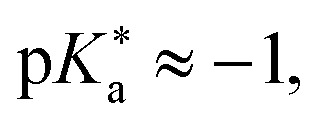
 whereas the p*K*_a_ of the conjugate acid of NMI is 7.4.^47^ In a previous study, we demonstrated that the bulk dielectric properties of the solvent system primarily account for dielectric stabilization without complications from specific solute–solvent interactions or dielectric enrichment effects.^48^ The dielectric constant of the mixture can be varied from *ε*_r_ = 6.0 to 24.8 while viscosity (*η* = 0.55 cP) and refractive index (*n* = 1.382) remain constant. In addition, the binary mixture does not disrupt the hydrogen-bonding equilibrium between the reaction partners thus being ideal for a systematic study of the dielectric stabilization.

Our primary objective is to examine the effect of the dielectric environment on ESPT from C_4_-dHONI to an external base NMI. We demonstrate that CIP* and SSIP*/FIP* can be spectrally resolved both in steady-state and time-resolved fluorescence spectra by deconstructing contributions from different species with the help of band-shape modeling. Time-resolved fluorescence additionally allows for monitoring the solvent stabilization that is manifested as time-dependent red shifts of the fluorescence bands. The fast kinetics resolved with broadband fluorescence up-conversion spectroscopy (FLUPS)^49^ appear similar in all solvent systems but both steady-state and ns-timescale fluorescence kinetics, investigated with time-correlated single photon counting (TCSPC), show distinct differences that are attributed to evolution of the ion pair species from SSIP* to FIP* upon increasing polarity. This hypothesis is supported by observing the formation of the fully separated ground-state ions by means of ns-transient absorption (ns-TA).

## 2 Experimental section

### 2.1 Materials and methods

Full experimental details of the spectroscopic methods are given in the ESI (Section S1).† The solvents, propyl acetate (≥99%, Sigma-Aldrich) and *n*-butyronitrile (≥99%, Sigma-Aldrich), were dried and stored over 3 Å molecular sieves under a nitrogen atmosphere. The dryness was verified prior to use by determining the *E*_T_(30) values of the neat solvents according to:

1 *E*_T_(30) [kcal mol^−1^] = *hcṽ*_0_*N*_A_ = 2.8591 × 10^−3^*ṽ*_0_ [cm^−1^],

where *ṽ*_0_ is the peak frequency of the low-energy absorption band of betaine-30 (B30). Deviation of the experimental *E*_T_(30) values was found to be ≤1% from the values reported in the literature (Table S1, ESI†).^48^ More details about the properties of the solvent mixtures are given in the ESI (Section S1.2).†

The solvent mixtures were chosen to have equal spacing in the reaction field factor, Δ*f*, defined as:^50^2
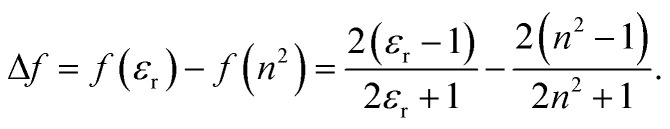
Many solvatochromic dyes exhibit a good linearity between the solvation energies and Δ*f*.^48^ Thus, the individual solvent mixtures are expected to be linearly spaced in solvation energy.

### 2.2 Fluorescence band-shape modeling

Prior to analysis, all fluorescence spectra were transformed into the so-called transition dipole moment (TDM) representation by dividing the corresponding spectra in wavelength by *ν̃*^5^, *i.e.*, *F*_TDM_(*ν̃*) ∝ *F*(*ν̃*)/*ν̃*^3^ = *F*(*λ*)/*ν̃*^5^.^51^ Here *F*(*λ*) is the distribution of fluorescence photons over wavelength, as recorded in our measurements. The TDM representation corresponds to the real lineshape of the underlying transition and does not get distorted upon spectral shifts. Furthermore, the areas of the band-shape functions of the time-resolved spectra are directly proportional to populations of the corresponding species scaled by their squared transition dipole moments (|*M*|^2^).^36^ For the present photoacid, the transition dipole moments of the protonated and deprotonated species (Scheme 1) are identical and thus the time-resolved band areas report directly on the relative populations.^27^

Steady-state and time-resolved fluorescence spectra were decomposed into contributions from the different emitting species. Up to three distinct bands could be resolved in the spectra and were attributed to ROH*, CIP* and SSIP*/FIP* according to Scheme 1. The spectral decomposition was achieved by fitting the spectra to a sum of one to three log-normal functions:^52,53^3
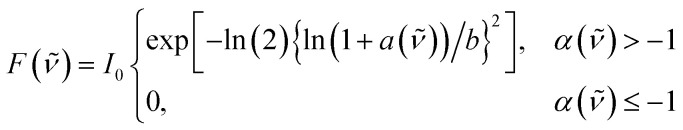
4
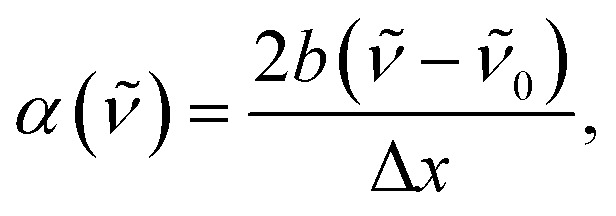
where *I*_0_ is the band intensity, *ν̃*_0_ the peak frequency, *b* the asymmetry parameter and Δ*x* the width parameter. Analytical expression for the full-width at half maximum (FWHM) and area of the log-normal function are given in the ESI (Section S3.1).†^53^ Full details of the other data analysis methods are given in the ESI.†

## 3 Results and discussion

### 3.1 Steady-state absorption and fluorescence spectra

**Fig. 1:**
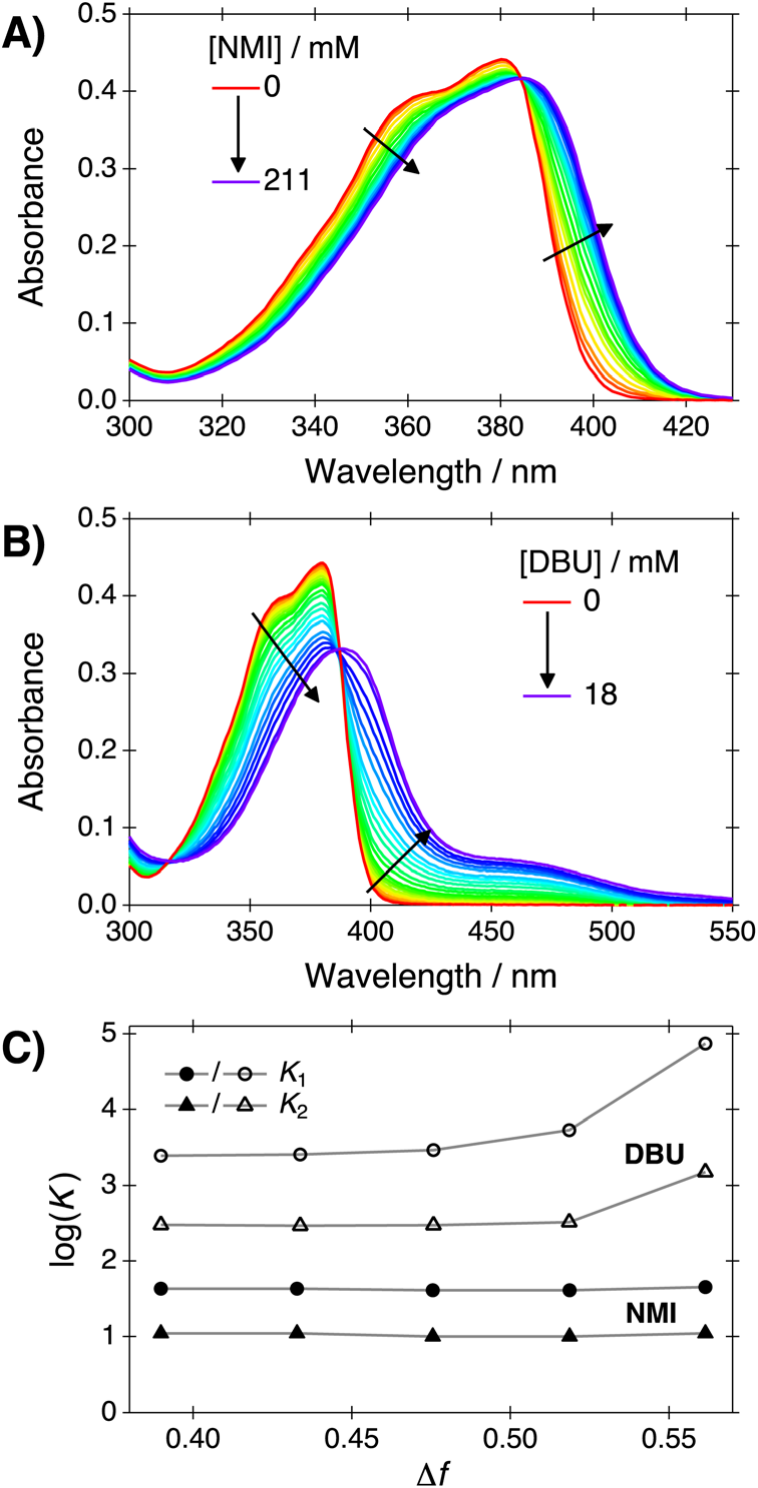
Steady-state absorption spectra of C_4_-dHONI (*c* = 30 μM) upon addition of (A) NMI and (B) DBU in a PA/BuCN solvent mixture (Δ*f* = 0.48). (C) Logarithmic association or deprotonation constants with NMI (solid markers) and DBU (empty markers) in all mixtures as a function of Δ*f*. The lines serve as a guide to the eye. Adapted from ref. 48.

In previous studies, we demonstrated that organic bases bind to the hydroxyl groups of C_4_-dHONI forming 1 : 1 and 1 : 2 complexes that can be identified in the absorption spectra.^30,48^ A weak base, NMI, hydrogen bonds to the hydroxyl groups of C_4_-dHONI, which is manifested as a slight broadening and a red shift of the main absorption band (Fig. 1A). Association with a strong base, DBU, on the other hand, results in deprotonation of a single –OH group and binding of another DBU on the other –OH group. The deprotonation is manifested in the absorption spectrum as a significant decrease and red shift of the main absorption band and an appearance of a new band centered at about 470 nm (Fig. 1B). This low-energy band becomes more prominent and red shifted upon increasing polarity whereas the spectral changes in the presence of NMI are comparable in all solvents (Fig. S3 and S4, ESI†).

Association constants for the formation of 1 : 1 and 1 : 2 complexes were determined from global analysis of the absorption spectra (Section S2, ESI†). The association constants with NMI are identical in all mixtures whereas a sudden increase is observed in the association constants with DBU at polarities above *ε*_r_ ≈ 10 (Δ*f* ≈ 0.48). The results demonstrate that the solvent mixtures do not interfere with the complex formation with NMI but the increased polarity will facilitate more efficient deprotonation. Furthermore, dissociation of the ground-state ion pair may become operative in the mixtures of highest polarity as suggested by the increased width and appearance of a low-energy tail in the absorption spectra in the presence of DBU (Fig. S4, ESI†).^30^

**Fig. 2:**
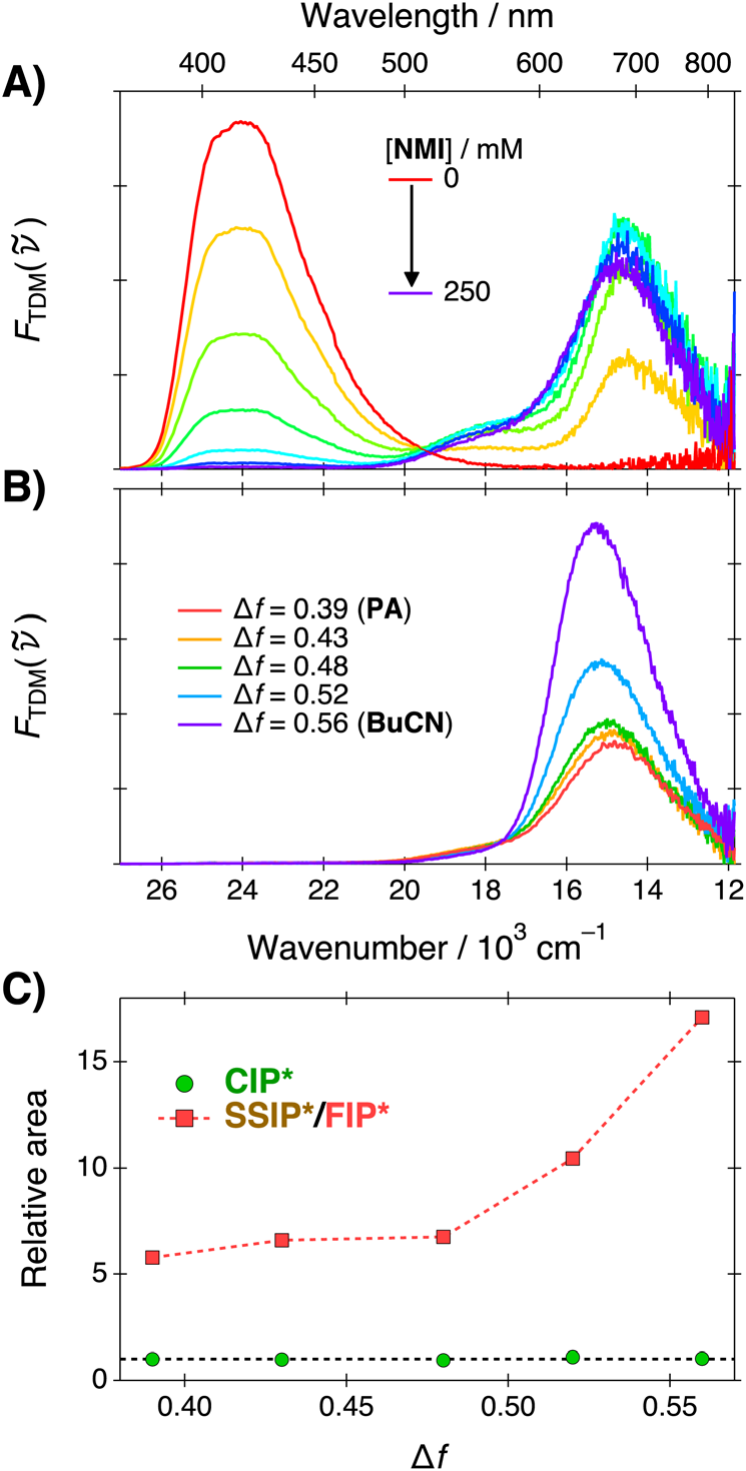
(A) Steady-state fluorescence spectra of C_4_-dHONI (*c* = 13 μM) upon addition of NMI in PA and (B) the spectra in all solvent mixtures in the presence of NMI (*c* = 250 mM). (C) Relative areas of CIP* and SSIP*/FIP* fluorescence bands normalized to mean CIP* area across all solvent systems.

Excitation of the ground-state complexes between C_4_-dHONI and NMI results in excited-state proton transfer and formation of ion-pair species. This is manifested in the fluorescence spectra as a disappearance of the neutral emission centered at around 415 nm and appearance of low-energy emission bands at above 500 nm. The fluorescence spectra upon addition of NMI in neat PA, presented in Fig. 2A, exhibit two distinct bands in the long-wavelength region centered at about 550 nm and 680 nm, which are attributed to CIP* and fully deprotonated (SSIP*/FIP*) species, respectively. Upon increasing solvent polarity, the CIP* band shifts towards red (positive solvatochromism) without significant change in its intensity whereas the lowest-energy band (SSIP*/FIP*) shifts towards blue (negative solvatochromism) and increases significantly in intensity (Fig. 2B). The negative solvatochromism of the SSIP*/FIP* band most likely arises from the large ground-state dipole moment induced by the negative charge that is localized on the deprotonated hydroxyl oxygen. In CIP, the charge is partly compensated by the bound protonated base.

Fluorescence band positions and areas were extracted by band-shape analysis using log-normal function, eqn (3) (see Section S3 for details and supplementary data, ESI†). Frequencies of the band maxima and full-width at half maxima (FWHM) are summarized in Table 1. The relative areas of the CIP* and SSIP*/FIP* bands, presented in Fig. 2C, remain nearly constant up to Δ*f* = 0.48 after which a significant enhancement of the SSIP*/FIP* band is observed. The increase in SSIP*/FIP* band intensity follows a similar trend to the deprotonation constant determined from the absorption spectra in the presence of DBU (Fig. 1C) and becomes more pronounced upon exceeding Δ*f* ≈ 0.48. Thus, the sudden enhancement of the SSIP*/FIP* band is most likely caused by the interplay between these ion pair species.

**Table 1:** Summary of the band-shape parameters of the different species obtained from the log-normal fitting of fluorescence spectra. The full-width at half maximum values are given in parenthesis. All values are given in 10^3^ cm^−1^

Δ*f*	ROH*	CIP*	SSIP*/FIP*
	*ṽ*_max_ (FWHM)	*ṽ*_max_ (FWHM)	*ṽ*_max_ (FWHM)
0.39	24.1 ± 0.3 (3.2)	17.5 ± 1.1 (3.3)	14.8 ± 0.8 (3.0)
0.43	24.0 ± 0.7 (3.2)	17.5 ± 1.4 (2.6)	14.9 ± 0.8 (3.0)
0.48	24.0 ± 0.4 (3.2)	17.4 ± 0.7 (3.1)	14.9 ± 0.7 (2.9)
0.52	23.9 ± 0.2 (3.2)	17.1 ± 0.9 (2.5)	15.1 ± 0.6 (2.8)
0.56	23.9 ± 0.8 (3.2)	16.9 ± 1.7 (2.8)	15.2 ± 0.9 (2.8)

### 3.2 Ultrafast ESPT dynamics

**Fig. 3:**
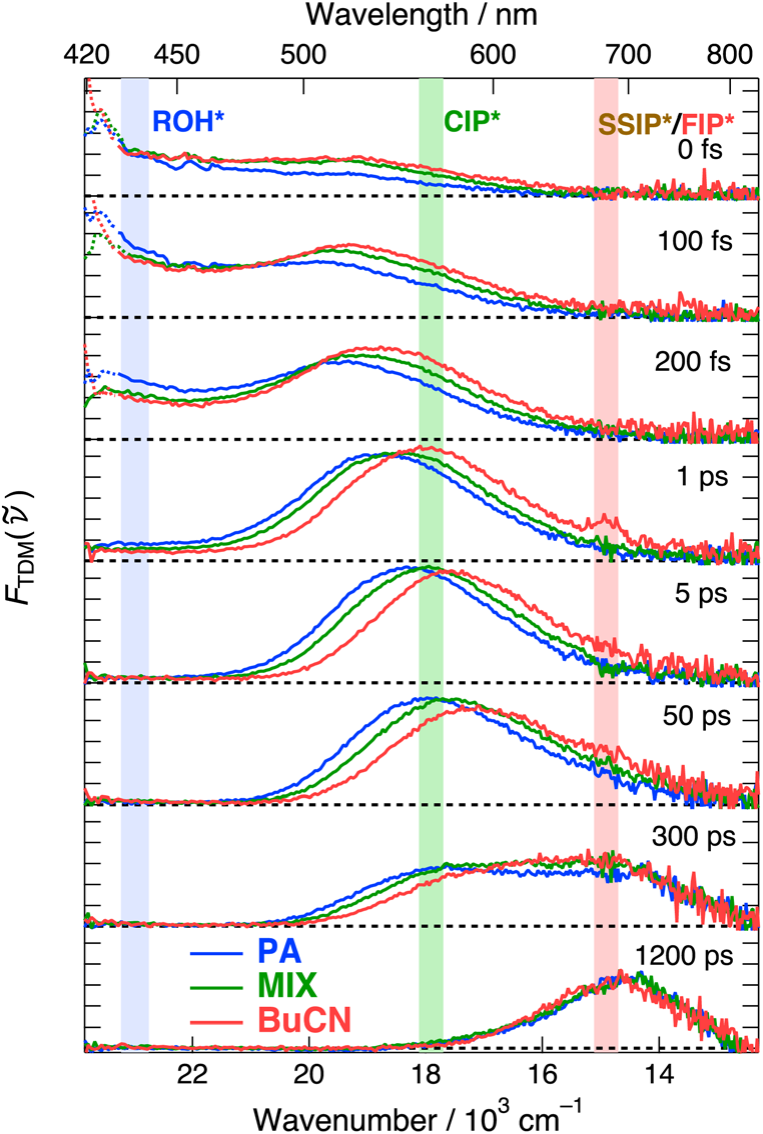
Time-resolved fluorescence spectra of C_4_-dHONI in the presence of NMI (*c* = 200 mM) in PA (blue), MIX (green) and BuCN (red). All sub-panels have the identical vertical scale. The colored vertical fills indicate the monitoring ranges for the respective species indicated in the top panel.

Ultrafast ESPT dynamics were monitored by means of broadband fluorescence up-conversion spectroscopy (FLUPS).^49^ The measurements were performed in three representative solvent systems corresponding to the neat solvents and one mixture with Δ*f* = 0.48 denoted as MIX. Representative FLUPS spectra at selected time steps are presented in Fig. 3. Further details and supplementary data are provided in the ESI (Section S4).†

Similarly to the steady-state fluorescence, three distinct fluorescence bands can be observed in the FLUPS spectra. Prompt ROH* emission is observed below 450 nm and a broad CIP* band above 500 nm. The structured peaks at around 420 nm and 450 nm are due to Raman scattering from the solvent due to inadequate subtraction of the solvent signal. The presence of the CIP* band at *t* = 0 fs indicates that ESPT is ultrafast and occurs partially within the experimental time resolution of *ca.* 190 fs. The ROH* band decays to nearly zero while the CIP* band reaches its maximum during the first few ps and the short-range ESPT is completed in about 5 ps. Nearly complete decay of the ROH* band indicates a unidirectional ESPT without reversibility. The CIP* band also undergoes a significant solvent relaxation in this time scale manifested as a dynamic red shift and reaches the fully relaxed spectrum in few tens of ps. The magnitude of the red shift scales almost linearly with Δ*f*, as expected based on our previous study.^48^ Finally, the CIP* band decays to zero on hundreds of ps time scale with a concomitant appearance of the long-wavelength emission band at around 700 nm attributed to the SSIP* and FIP* species. Again, the complete decay of the CIP* band suggests a unidirectional reaction. The decay of the CIP* band and appearance of the SSIP*/FIP* band is completed within the experimental time window of 1.2 ns but the decay of the SSIP*/FIP* band occurs on a significantly longer time scale.

**Fig. 4:**
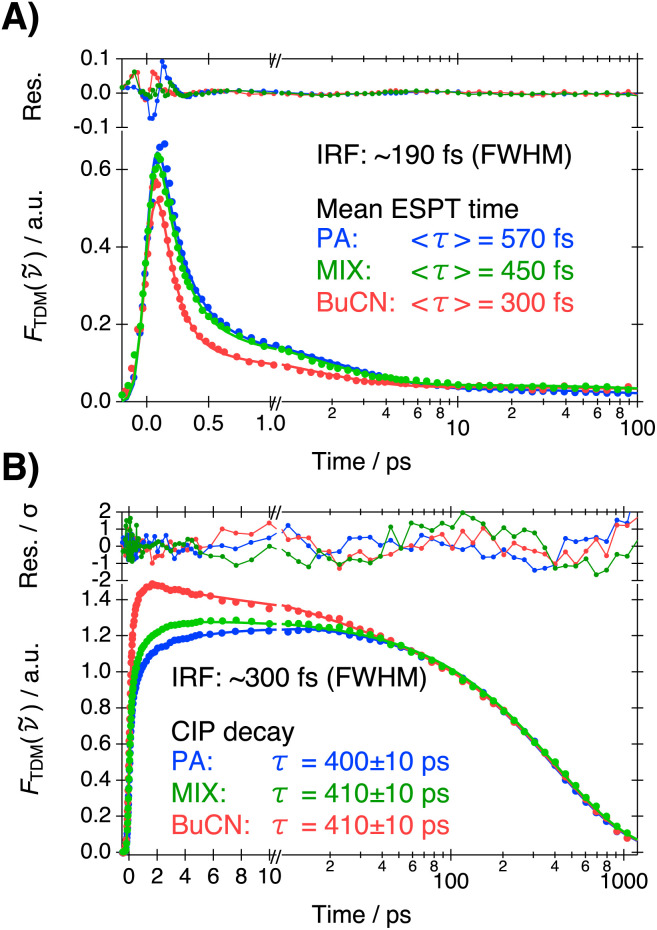
Fluorescence decays of (A) ROH* and (B) CIP* in PA (blue), MIX (green) and BuCN (red) together with multi-exponential fits and residuals. The intensity of the ROH* band corresponds to the total area obtained from the band-shape modeling whereas the decay of the CIP* form corresponds to the averaged intensity from the green vertical fill indicated in Fig. 3. The mean ESPT times are given in the inset of (A). The CIP decay times given in (B) correspond to the longest-lived component of the exponential fits.

The time-resolved fluorescence spectra were analyzed by a time-dependent band-shape analysis where the spectra were modeled by a sum of two to three log-normal functions at each time step.^27^ The resulting band areas were analyzed by multi-exponential functions to extract the population dynamics whereas the solvent relaxation was analyzed from the time-dependent band positions. The ESPT dynamics were monitored from the decay of the ROH* band and analyzed using three-exponential functions. The ROH* band areas and exponential fits are presented in Fig. 4A and lifetimes and amplitudes are given in Table 2.

**Table 2:** Lifetimes and amplitudes from the multi-exponential fits of the time-resolved fluorescence bands of the ROH* and CIP* forms together with mean solvation times. All lifetimes are given in ps

Δ*f*	ROH* decay					〈*τ*_solv_〉^b^
	*a*_1_	*τ*_1_	*a*_2_	*τ*_2_	〈*τ*_ave_〉^a^	
0.39	0.84	0.21	0.16	2.4	0.57	2.3
0.48	0.83	0.18	0.17	1.7	0.45	3.9
0.56	0.88	0.14	0.12	1.6	0.30	0.8

a The average lifetime is calculated as 〈*τ*_ave_〉 = Σ*α*_i_*τ*_i_.

b Mean solvation times from ref. 48.

Acceleration of the ESPT dynamics upon increased polarity (Δ*f*) is clearly visible in Fig. 4A. In all solvent systems, majority (>80%) of the population decays with a time constant that is close to the IRF (*ca.* 190 fs). Therefore, a significant fraction of the population decays within the IRF and the total amplitude does not reach unity at *t* = 0. The lifetime of the fastest component becomes shorter and amplitude larger upon increasing polarity. The fast decay is followed by a ps decay component that also becomes faster upon increasing polarity. In all solvents, a minor (<4%) long-lived component extending up to hundreds of ps is observed and likely results from an uncomplexed photoacid. The mean ESPT time calculated from the two fastest time constants is reduced from 570 fs in PA to 300 fs in BuCN.

The rise and decay of the CIP* form are less accurately captured in the time-dependent band shape analysis (see Section S4, ESI†). The rise does become significantly faster in BuCN but the trend is less clear (Table S6, ESI†) due to uncertainties in the IRF-limited rise components. Therefore, the ESPT dynamics are more accurately reported by the decay of the ROH* form.

Two main conclusions can be drawn from the ROH* decays. First, the initial ESPT is significantly faster than solvation, in contrast to what is usually observed in ESPT to solvent.^27^ The fastest time constant for the solvation in these mixtures is about 250 fs, accounting for *ca.* 55% of the solvent stabilization.^48^ This demonstrates that the initial ESPT in the preformed hydrogen-bonded complex does not require significant solvent reorganization. The lack of solvent control in the initial step also explains why bimolecular ESPT can proceed in nonpolar solvents, as has been reported in the literature.^54^ The second conclusion pertains to the slower decay component that however occurs within the solvent relaxation but becomes more significant upon decreasing polarity. This is likely due to a distribution of initial hydrogen-bonded geometries requiring a different extent of solvent reorganization for ESPT to occur. This bears similarity to ESPT to solvent, where the reaction is largely driven by reorganization of the accepting solvent.^27^

After the ultrafast ESPT and solvent relaxation the CIP* band decays with a concomitant appearance of the SSIP*/FIP* band. CIP* and SSIP*/FIP* bands exhibit significant overlap in the hundreds of ps time scale and could not be fully separated by band-shape modeling with meaningful constraints on the fitting parameters. Furthermore, the noise drastically increases in the red because of the TDM representation (division by *ν̃*^5^). Due to these reasons, decay of the CIP* band was monitored at around 560 nm, as indicated by the green vertical fill in Fig. 3. The decay traces together with the multi-exponential fits and weighted residuals are presented in Fig. 4B. The lifetimes and amplitudes are summarized in Table S7 (ESI).†

The fast rise during the first few ps reflects the initial ESPT process, being significantly faster in a more polar solvent, but is also contaminated by the spectral shifts due to solvent relaxation. The decay of the CIP* form, on the other hand, occurs after solvent relaxation and reflects the dissociation of CIP* into SSIP*. Interestingly, the decay follows identical dynamics in all solvents with a time constant of *ca.* 400 ps. A comparable time constant could also be extracted from the band-shape modeling (Table S6, ESI†). This demonstrates that the dissociation is purely governed by viscosity as expected for a diffusion-controlled process.

However, the observed 400 ps time constant corresponds to the total decay rate of the CIP* form. If the intrinsic lifetime of the CIP* form is comparable to that of the FIP* (>10 ns, *vide infra*), over 90% of the relative population would dissociate into SSIP*/FIP*. The band-shape modeling, however, demonstrates that only about 50% of the population is transformed into SSIP*/FIP* (Fig. S14, ESI†). Therefore, almost half of the population decays directly from the CIP* to the ground state *via* an additional quenching pathway. Moreover, the time constant for the dissociation is about two times larger than the observed decay time. Based on our previous study, the most probable quenching mechanism is a proton recombination. This is supported by the efficient fluorescence quenching observed at low pH.^47^

### 3.3 Long timescale fluorescence dynamics

**Fig. 5:**
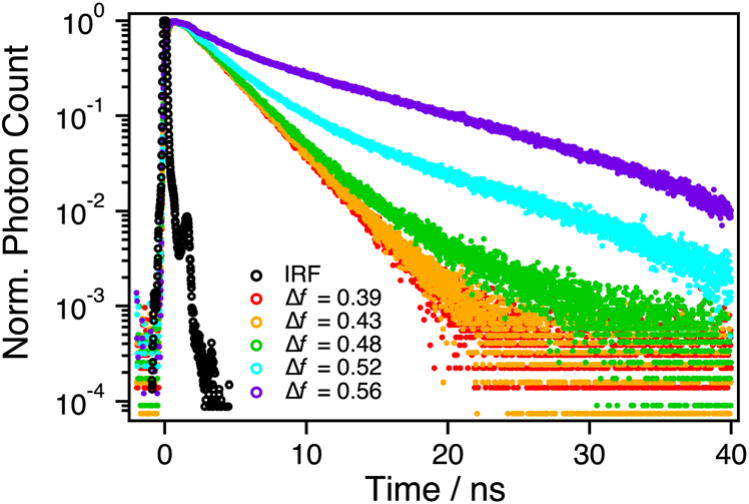
TCSPC fluorescence decays of C_4_-dHONI in the presence of NMI (*c* = 220 mM) monitored at 670 nm upon 375 nm excitation in the binary mixtures.

Evolution of the SSIP*/FIP* forms occurs on the ns-timescale and was investigated by TCSPC. Fluorescence dynamics of these species were monitored at around 670 nm indicated by the red vertical fill in Fig. 3. The decays, presented in Fig. 5, were analyzed by two- or three-exponential functions and the lifetimes and amplitudes are given in Table 3. Further details and supplementary data are provided in the ESI (Section S5).†

**Table 3:** Lifetimes, amplitudes and *χ*_r_^2^ values obtained from the fitting of the fluorescence decays monitored at 670 nm upon 375 nm excitation

Δ*f*	*a*_1_	*τ*_1_/ns	*a*_2_	*τ*_2_/ns	*a*_3_	*τ*_3_/ns	*χ*_r_^2^
0.39	Rise	0.37	1	2.7	—	—	1.12
0.43	Rise	0.36	1	2.7	—	—	1.13
0.48	Rise	0.38	0.98	2.8	0.02	7.0	1.18
0.52	Rise	0.35	0.79	2.8	0.21	10.1	1.17
0.56	Rise	0.39	0.53	2.7	0.47	12.1	1.31

The fluorescence decays exhibit a fast rise of about 0.4 ns in all solvents that can be attributed to dissociation of the CIP* into SSIP*/FIP*, in agreement with the FLUPS measurements. A major decay component of about 2.7 ns is observed in all solvent systems but another component with a *ca.* 10 ns time constant appears in polar solvents. Again, the appearance of this component coincides with the sudden increase in the steady-state fluorescence of the SSIP*/FIP* forms above Δ*f* = 0.48. Based on our data, we attribute the 2.7 ns component to SSIP* and *ca.* 10 ns component to FIP*. Full dissociation from SSIP* into FIP* is facilitated by the increased polarity above Δ*f* = 0.48 whereas the population is trapped as SSIP* below this threshold. The much longer lifetime of the FIP* form results in significant enhancement of the long-wavelength fluorescence band explaining the variation in the steady-state fluorescence. The differences in deprotonation constants and absorption spectra observed upon addition of a strong base (DBU) suggest that the dissociation is also operative in the ground state in the same polarity range, although the yield is likely much smaller.

### 3.4 Formation of ground-state ion population

**Fig. 6:**
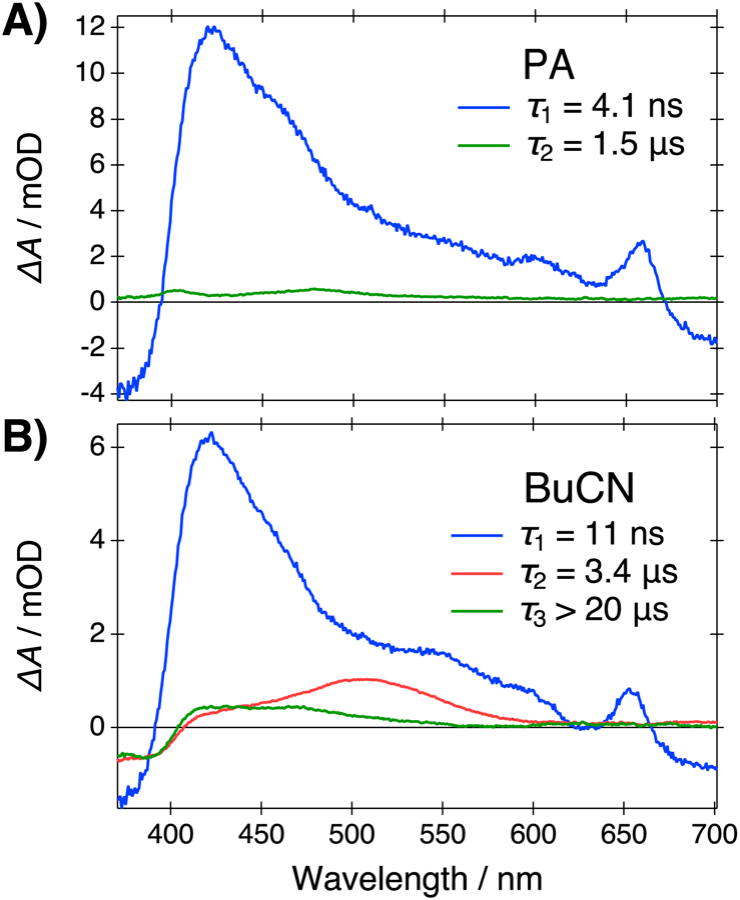
Evolution Associated Difference Spectra (EADS) and associated lifetimes resulting from the global analysis of the ns-TA data in (A) PA and (B) BuCN. Samples were excited at 355 nm.

Dissociation into the FIP* form is expected to have a drastic effect on the recombination dynamics in the ground state. The ground-state SSIP form is expected to recombine into CIP and subsequently convert to ROH relatively fast due the presence of the Coulomb interaction between the ion pair. Therefore, we expect that a significant ground-state population of the deprotonated acid is produced only upon formation of the FIP* form, *i.e.*, at Δ*f* > 0.48. To test this hypothesis, we monitored the ground-state population by measuring the ns-transient absorption spectra in the same solvent systems. The time-resolved spectra were analyzed globally using a sequential model to extract the Evolution Associated Difference Spectra (EADS) and associated time scales. Representative EADS in PA and BuCN are presented in Fig. 6. Further details and supplementary data are provided in the ESI (Section S6).†

In all solvents, the decays exhibit a fast initial component (blue in Fig. 6) with a characteristic excited-state absorption band (ESA) at around 430 nm, ground-state bleach (GSB) below 400 nm and stimulated emission above 600 nm that can be attributed to the excited-state anion (SSIP*/FIP*).^55^ The initial deprotonation and decay of the CIP* form are not resolved due to the limited time resolution. The lifetime of the initial component increases significantly above Δ*f* = 0.48 and in BuCN it is comparable to the fluorescence lifetime of the FIP* form.

The fast initial component is followed by a very long-lived species (green in Fig. 6) extending up to several μs in all solvent systems. At Δ*f* ≤ 0.48, only these two components are resolved. Above Δ*f* = 0.48 an additional intermediate component (red in Fig. 6) with an ESA band at around 500 nm appears. Its amplitude and lifetime significantly increase upon increasing polarity. Based on the spectral signature and the long lifetime, this species can be attributed to the fully dissociated ground-state anion (FIP).^56^ In agreement with our hypothesis, the fully dissociated ions are produced selectively in polar solvents above Δ*f* = 0.48 with a yield that depends strongly on the polarity. Comparison of the spectral signature of the deprotonated ground-state species with the steady-state spectra in the presence of a strong base (Fig. 1) provides further support for this conclusion. The main absorption band of the deprotonated species in the presence of a strong base appears at around 470 nm, most likely representing the hydrogen-bonded or contact ion pairs, whereas the relatively weak long-wavelength tail appearing in BuCN (Fig. S4, ESI†) indicates the formation of the solvent-separated or fully dissociated ions.

The nature of the other long-lived species observed in all solvents is less clear although a similar long-lived component has been observed before for related compounds.^56^ The spectral signature of this component with two distinct ESA bands at around 400 nm and 470 nm (Fig. S19, ESI†) is spectrally similar to that of the radical anion reported for *t*Bu-substituted 1,8-naphthalimides. Therefore, the observed component probably corresponds to the radical anion of the protonated photoacid generated *via* an electron transfer process from the organic base. In support of this hypothesis, quenching of the total fluorescence was observed at high base concentrations indicating the presence of an additional deactivation pathway with increased efficiency at high base concentration. However, the detailed nature of this species was not investigated further in our study.

### 3.5 Influence of solvent properties on ion-pair dynamics

Ion pair dynamics between C_4_-dHONI and NMI have been previously investigated by TCSPC in acetonitrile (MeCN) and benzonitrile (PhCN).^30^ Both of these solvents are more polar than the present solvent mixtures, but MeCN is less viscous (*η* = 0.34 cP) whereas PhCN is more viscous (*η* = 1.24 cP). Comparison with the current data provides deeper insights into the influence of different macroscopic solvent properties on the ion pair dynamics. Dissociation of the CIP* into SSIP* depends strongly on the viscosity, being significantly faster in MeCN (*τ*_CIP*_ = 160 ps) and significantly slower in PhCN (*τ*_CIP*_ = 720 ps). Furthermore, the present results demonstrate that the polarity has no impact on the initial diffusion controlled separation from CIP* into SSIP* in this polarity range. The energy gain from the solvation energy is large enough for the dissociation of the contact ion pair. We anticipate that this observation can be generalized to other strong photoacids where the hydrogen bond between the deprotonated acid and the protonated base is extremely weak due to the significant charge transfer character of the S_1_ state of the photoacid. However, separation of the CIP* may become less facile in weaker photoacids with weaker charge transfer character and in nonpolar solvents with smaller solvation energies. For example, the well-known HPTS (8-hydroxypyrene-1,3,6-trisulfonate) has been shown to remain hydrogen bonded to a protonated base in nonpolar benzene^54^ whereas full separation occurs in more polar NMI.^57^ The time scale for the full dissociation into FIP* appears to also depend on the viscosity, being significantly slower in PhCN (*τ*_SSIP*_ = 4.4 ns). However, the dynamics in MeCN (*τ*_SSIP*_ = 2.9 ns) are comparable to the present solvent systems (*τ*_SSIP*_ = 2.7 ns) but the yield of the FIP* clearly correlates with the polarity. A significantly higher (>80%) relative amplitude of the fluorescence decay component attributed to FIP* was observed in MeCN.^30^

It also interesting that the SSIP* exhibits a constant decay rate in the present solvent systems. Full separation into FIP*, being operative only at high polarity, would be expected to accelerate the decay of the SSIP* form. The constant decay rate thus suggests that other processes must also contribute to the overall decay. Since the transition dipole moments of the different ion pair species are identical, the radiative rates of the SSIP* and FIP* forms are also expected to be the same.^27^ However, the lifetime of the SSIP* (2.7 ns) is about four times shorter than that of the FIP* (12.1 ns). This demonstrates that the decay of the SSIP* is dominated by SSIP* → GS decay which, similarly to the CIP*, is probably induced by the proton recombination. Since both the proton recombination and dissociation are diffusion-controlled processes, the SSIP* decays with a constant rate in the present solvent system. This also explains the longer SSIP* decay time in the more viscous PhCN. The yield of the FIP* is nevertheless dictated by the polarity of the solvent system.

## 4 Conclusions

The present results provide a comprehensive picture of dissociation of strong acids in organic solvents of intermediate polarity. Bimolecular ESPT from a strong photoacid to an organic base occurs in a step-wise manner through several intermediates with dynamics and yields controlled by the macroscopic solvent properties. Initial ESPT in a preformed hydrogen-bonded complex is ultrafast, approaching the time scale of the fastest intramolecular ESPT reactions. ESPT in such complexes occurs significantly faster than solvent relaxation thus requiring minimal solvent reorganization and is expected to proceed even in a nonpolar environment. However, such hydrogen-bonded complexes exist as a distribution of geometries that require different amounts of reorganization to reach the reactive state. Since the absorption spectrum of the complex depends on the strength of the hydrogen bond, the initial ESPT dynamics are expected to be dependent on the excitation wavelength that can selectively excite a sub-population of the overall system.

Initial ESPT in these complexes produces contact ion pairs that further dissociate into solvent-separated ion pairs with dynamics that, in solvents of intermediate polarity, depend solely on the viscosity. This is attributed to the extremely weak hydrogen bond between the reactant pair and significant solvation energy of the generated ions, which results from the strong charge-transfer character of the excited state of strong photoacids. However, the dissociation might be hindered for weaker photoacids, being a trade-off between the hydrogen-bond strength and the solvation energy. The solvent-separated ion pairs may further dissociate into free ion pairs but this occurs only in polar solvents with *ε*_r_ > 10. The time scale of the dissociation shows a weak dependence on viscosity, being somewhat slower in high viscosity solvents, but the yield of the free ion pairs increases significantly upon increasing polarity. In low polarity solvents with *ε*_r_ < 10, full dissociation does not occur and the population is trapped as solvent-separated ion pairs. This has a direct impact on the yield of the ground-state ions. Significant ground-state population of dissociated ions is produced only upon full dissociation into free ion pairs in the excited state. The intermediate ion pair species recombine too fast preventing the escape of the ions from the Coulomb well. Due to the spectral similarity between the solvent-separated and free ion pairs, determination of the yield of ground-state ions purely from fluorescence can result in erroneous conclusions.

Our results provide guidelines for optimizing the solvent properties of organic solvents of intermediate polarity in systems where generation of ions is desired for the final application. Furthermore, our results could be of interest for testing statistical mechanics theories due to the broad range of dynamic parameters obtained upon the systematic variation of a single macroscopic solvent parameter.

## Author contributions

The work was conceived and supervised by TSK. AR, PV and TSK performed the spectroscopic experiments and analyzed the data. AB synthesized the compounds. PM assisted with the femtosecond instruments. AR and TSK wrote the manuscript.

## Conflicts of interest

There are no conflicts to declare.

## Supplementary Material

SC-016-D5SC03404C-s001

## Data Availability

Data supporting this article have been included as part of the ESI.†
