# Supplementary material for: Dielectric stabilization controls excited-state proton transfer and ion pair dynamics in organic solvents
Source: Chem Sci. 2025 Jun 30;16(30):13935–43. doi: 10.1039/d5sc03404c (PMC12230855; doi:10.1039/d5sc03404c)
Supplement: SC-016-D5SC03404C-s001 [file SC-016-D5SC03404C-s001.pdf]

Supplementary Information (SI) for Chemical Science.
This journal is © The Royal Society of Chemistry 2025

Dielectric stabilization controls excited-state proton transfer and ion pair dynamics in organic solvents

---

Electronic Supplementary Information

Amar Raj,[†] Pragya Verma,[‡] Andrei Beliaev,[†,¶] Pasi Myllyperkiö,[†] and Tatu Kumpulainen[*,†]

†*Department of Chemistry/Nanoscience Center, University of Jyväskylä, P.O. Box 35, Jyväskylä, FI-40014, Finland*

‡*Department of Physical Chemistry, University of Geneva, 30 Quai Ernest Ansermet, Geneva, Switzerland.*

¶*Present address: Department of Chemistry, University of Eastern Finland, Yliopistokatu 7, FI-80101 Joensuu, Finland.*

E-mail: tatu.s.kumpulainen@jyu.fi

Contents

S1 Experimental section **S3**

S1.1 Materials S3

S1.2 Solvent mixtures S3

S1.3 Steady-state spectroscopy S4

S1.4 Broadband Fluorescence Up-Conversion Spectroscopy (FLUPS) S4

S1.5 Time-correlated single photon counting (TCSPC) S7

S1.6 Nanosecond transient absorption (ns-TA) spectroscopy S8

S2 Ground-state association and deprotonation constants **S9**

S3 Steady-state spectra and band-shape analysis **S14**

S3.1 Band-shape analysis of the steady-state fluorescence spectra S16

S4 Broadband fluorescence up-conversion spectra **S23**

S4.1 Band-shape analysis of the FLUPS spectra S25

S5 Nanosecond fluorescence decays (TCSPC) **S32**

S6 Nanosecond transient absorption (ns-TA) **S35**

References **S41**

S1 Experimental section

S1.1 Materials

The photoacid (**C$_4$-dHONI**) was synthesized and purified following the previously reported procedures.[1] The bases *N*-methylimidazole (**NMI**, $\geq$ 99%, TCI) and 1,8-diazabicyclo[5.4.0]-undec-7-ene (**DBU**, $\geq$ 98%, TCI) were used as received.

S1.2 Solvent mixtures

The solvents, propyl acetate (**PA**, $\geq$ 99%, Thermo Scientific) and butyronitrile (**BuCN**, $\geq$ 99%, Thermo Scientific) were dried over 3 Åmolecular sieves under nitrogen atmosphere. A Schlenk line was utilized for storing and handling the neat solvents.

The dryness of the solvents was assessed from the UV-vis absorption spectrum of betaine-30 (**B30**). **B30** is a highly sensitive solvatochromic dye, that responds to variations in polarity and hydrogen-bonding affinity of the solvent and is also very sensitive to solvent impurities.[2] The $E_\mathrm{T}(30)$ values values of the neat solvents were determined according to:

$$E_\mathrm{T}(30)\,[\mathrm{kcal/mol}] = hc\tilde{v}_0 N_\mathrm{A} = 2.8591 \cdot 10^{-3}\,\tilde{v}_0[\,\mathrm{cm}^{-1}], \tag{S1}$$

where $\tilde{v}_0$ is the peak frequency of low-energy absorption band of betaine-30 (B30). The determined $E_\mathrm{T}(30)$ values in **PA** and **BuCN** were 37.7 (37.5) kcal/mol and 42.6 (42.5) kcal/mol, respectively, in good agreement with the literature values given in parenthesis.[3,4]

The dielectric constant, ϵ_r, of the binary mixtures increases from 6.0 to 24.8 upon going from neat **PA** to neat **BuCN** and varies linearly with the weight fractions of the solvents according to:

$$\epsilon_r = w_\mathrm{PA} \cdot \epsilon_r(\mathrm{PA}) + (1 - w_\mathrm{PA}) \cdot \epsilon_r(\mathrm{BuCN}), \tag{S2}$$

where w_PA is the weight fraction of **PA**, whereas viscosity ($\eta = 0.55\,\mathrm{cP}$) and refractive index ($n = 1.382$) remain constant. The solvent mixtures were selected to be equally spaced in the reaction field factor, Δf, defined as:

$$\Delta f = f\left(\varepsilon_\mathrm{r}\right) - f\left(n^2\right) = \frac{2\left(\varepsilon_\mathrm{r} - 1\right)}{2\varepsilon_\mathrm{r} + 1} - \frac{2\left(n^2 - 1\right)}{2n^2 + 1}. \tag{S3}$$

The neat solvents, **PA** and **BuCN**, and three binary mixtures were selected for the study. Many solvatochromic dyes exhibit a good linearity between the solvation energy and Δf.[2] Thus, the individual solvent mixtures are expected to be linearly spaced in solvation energy. All relevant solvent parameters of the mixtures are given in Table S1.

Table S1: Parameters of the binary mixtures used

Δf	w_{BuCN}	w_{PA}	$\varepsilon_r{}^a$	$E_T(30)^a$	η^a	n^a	α^b	β^b	π^{*b}
0.39	0.00	1.00	6.0	37.5	0.581	1.382	0.00	0.40	0.53
0.43	0.08	0.93	7.5	39.0	0.581	1.382	-	-	-
0.48	0.21	0.79	10.0	40.3	0.581	1.382	-	-	-
0.52	0.50	0.50	14.3	41.4	0.581	1.382	-	-	-
0.56	1.00	0.00	24.8	42.6	0.581	1.382	0.00	0.45	0.63

[a]Dielectric constant ε_r, $E_T(30)$ (in kcal/mol), dynamic viscosity η (in cP) and refractive index n correspond to experimentally determined values from ref. 2. [b]α, β, and π^* are the Kamlet-Taft parameters related to hydrogen-bond (HB) donating, HB accepting ability and dipolarity/polarizability, respectively, taken from ref. 4.

S1.3 Steady-state spectroscopy

Absorption spectra were recorded with a Perkin Elmer Lambda 650 UV-vis spectrophotometer. Fluorescence spectra were recorded with a Varian Cary Eclipse spectrofluorometer corrected for the spectral sensitivity using a set of secondary emissive standards.[5] All measurements were performed at room temperature (22 ± 1°C) using a 1 cm quartz cuvette.

S1.4 Broadband Fluorescence Up-Conversion Spectroscopy (FLUPS)

The experimental setup for recording time-resolved fluorescence in this study is based on the optical design of Ernsting.[6–8] The corresponding schematic is shown in Figure S1. Initially, pump pulses at 399 nm are generated by frequency doubling part of the fundamental output from a 1 kHz Ti:sapphire amplified system (Coherent, Astrella). This pump beam is directed through a half-wave plate and a polarizer to adjust the polarization and final excitation power. The polarization was set to the magic angle, ensuring exclusive detection of population dynamics, and the excitation power was approximately 0.30 mW ($E_{pulse} = 0.30$ μJ). The pump beam is imaged through a 200 μm pin hole 2:1 onto the sample to improve the mode of the excitation spot with a final diameter of about 100 μm (full beam waist) at the sample.

Fluorescence from the excitation spot is then collected by an off-axis Schwarzschild objective and directed onto a calcite prism (with the optical axis out of the plane), isolating the vertically polarized component. Pump transmission is reduced by a beam stop on the Schwarzschild objective and a long-pass filter in front of the calcite prism. The fluorescence spot is imaged from the calcite prism on the non-linear crystal where it is overlapped with a horizontally polarized 1345 nm gate pulse, generated using the remaining portion of the fundamental output in an optical parametric amplifier.

Up-converted fluorescence is produced by non-collinear type II sum frequency generation

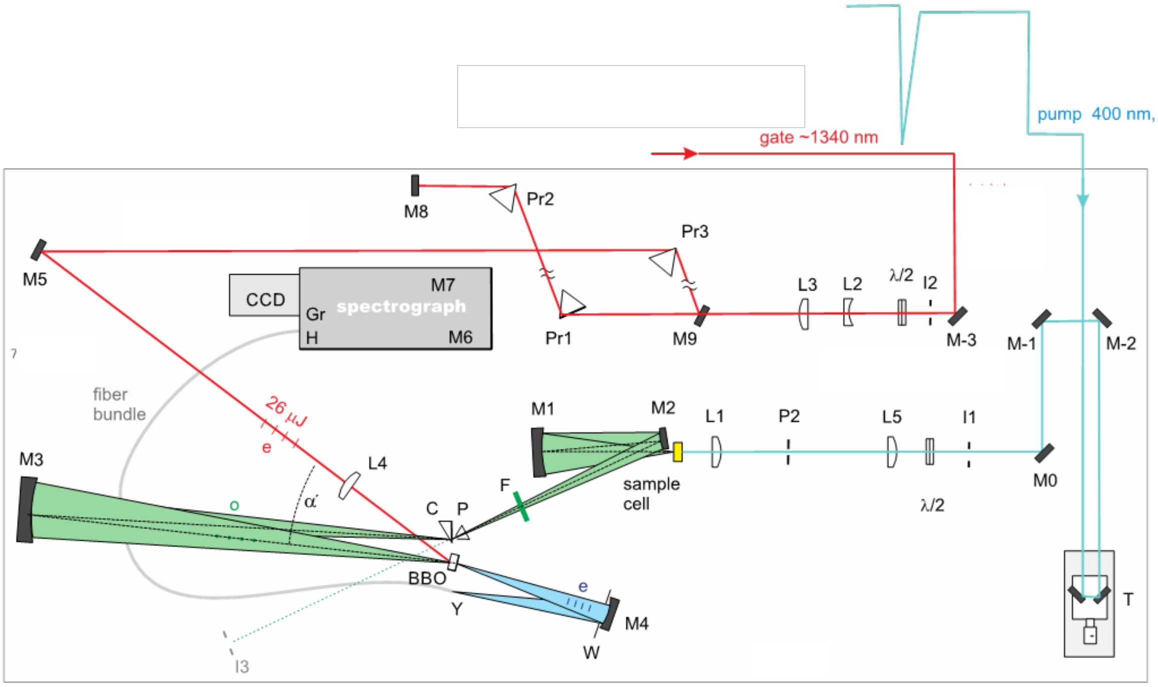

Figure S1: Schematic representation of the FLUPS setup used in this study. Main the beam paths are given by the colored lines and key components are represented by the following abbreviations: mirrors (M), prisms (Pr), lenses (L), half-wave plates ($\lambda/2$), calcite prism (C), translation stage (T), pin holes (P) and irises (I). The pump beam is frequency doubled to 400 nm from the fundamental frequency of the amplified laser system, while the gate at 1340 nm is generated in a home-built OPA. The up-converted signal is imaged onto an optical fiber and guided to a custom-built spectrograph where the signal is dispersed and detected by a CCD camera.

in a 0.1 mm thick BBO crystal, with the optical axis oriented in the horizontal plane in "case A" phase matching orientation.[8] The resulting signal is dispersed in a custom-built spectrograph and captured using a CCD camera (iDus DV420A-BU, Andor). The dynamics of the fluorescence signal are recorded using a computer-controlled delay stage in the pump path. The delay stage for the pump path allows scanning the time delay up to 1.2 ns.

A large beam-crossing angle of approximately 21.5° between the fluorescence and the gate beam enables broad phase matching and background-free signal detection. The large angle affects the temporal resolution of the experiment due to pulse-front shearing. To mitigate this effect, the gate pulse front is tilted using a single N-SF66 prism, which is then magnified by a focusing lens to match the pulse front of the fluorescence. Furthermore, a prism-compressor (N-SF66 prisms cut at the Brewster angle) is used to compress the gate pulses before the pulse front tilt is introduced. The time resolution is estimated by measuring the full width

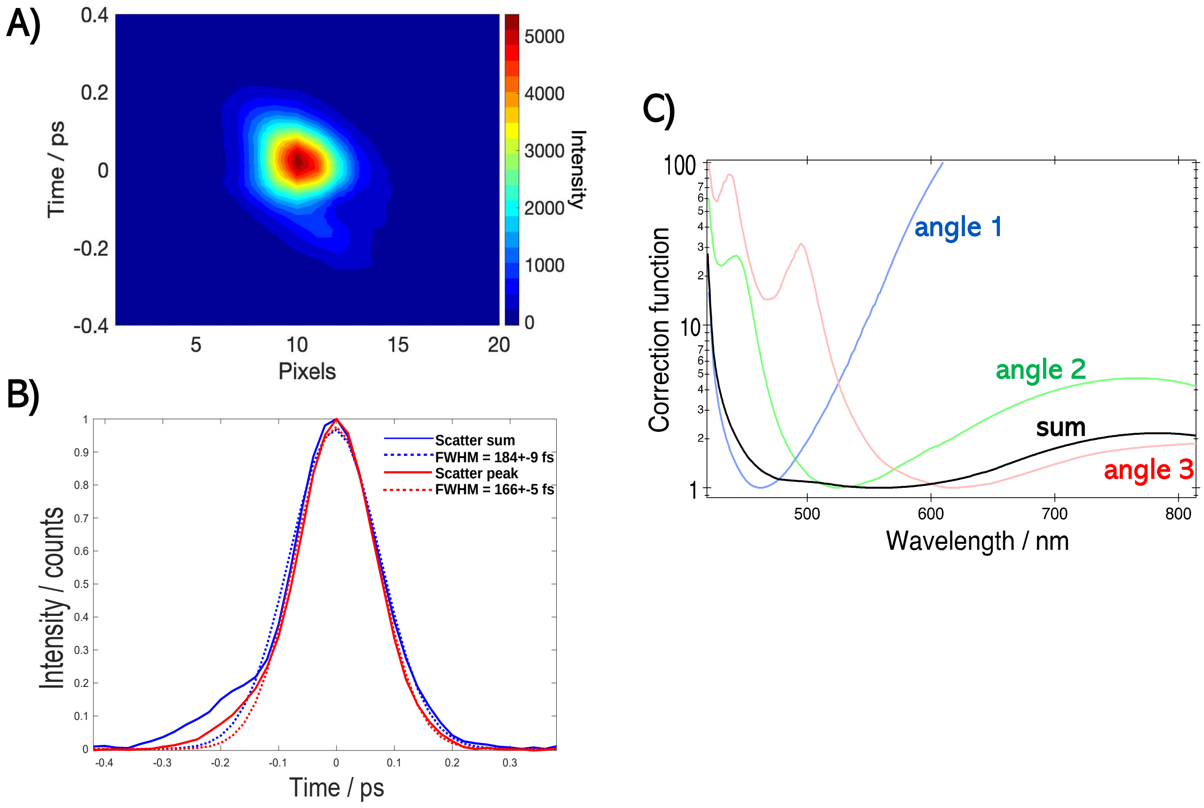

Figure S2: A) Up-converted cross-correlation signal of the pump and gate pulses. B) Gaussian-modeled cross-correlation at the peak maximum (red) and of the integrated signal (blue). The solid lines represent the experimental data and the dashed lines the Gaussian fits. C) Photometric correction functions measured at the individual crystal angles and that corresponding to the sum of all angles. The black represents the sum and the blue, green, and red represent the individual crystal angles.

at half maximum (FWHM) of the temporal cross-correlation between the pump and gate pulses presented in Figure S2A. The cross-correlation signal was integrated over the whole pixel range and modeled using a Gaussian function. The typical FWHM value of our setup is 190±20 fs as shown in Figure S2B.

The FLUPS signal was recorded at three different crystal angles with detuning of ±2 degrees around the central angle to increase the phase matching efficiency and signal-to-noise (S/N) over the whole detection range of about $14 \cdot 10^3$ cm^{-1}. The full procedure, including an interlaboratory comparison of the photometric accuracy was published recently.[9] The photometric correction functions were generated using a set of reference dyes: **BBOT**, **C6H**,

C153, **DCM**, and **LDS751** in dry acetonitrile.[5] The final correction function was generated from the sum of the signals measured at the three crystal angles. A polynomial fit was applied to the final correction function to remove noise. Photometric correction functions collected at the individual crystal angles and the correction function resulting from the sum of the three angles are presented in Figure S2C.

The chirp caused by the group velocity dispersion was independently measured from the instantaneous signal rise of a BBOT reference sample in the same sample cell and solvent under identical experimental conditions as the sample measurement. The t_0 values at each wavelength were obtained by fitting the rising signal with an exponential function convolved with a Gaussian simulated IRF representing the excitation pulse. These t_0 values were further extrapolated over the entire detection range using a chirp polynomial in the form of

$$t_0(\lambda) = a_1 + \frac{a_2}{\lambda^2} + \frac{a_3}{\lambda^4}, \tag{S4}$$

where λ corresponds to the fluorescence wavelength.

The data collection and processing involved several steps. First, signal from the sample and a solvent blank were measured at the three different crystal angles. Cosmic spikes were removed and 15 to 30 scans with 1 s integration time at each time step were averaged for the sample at each crystal angle and 3 scans were averaged for the blank. The averaged blank was subtracted from the signal at each crystal angle and the subtracted signals were summed up to yield the final fluorescence distribution as a function of CCD pixels. Pixels were converted to wavelength using a wavelength calibration of the CCD, determined from the atomic emission lines of a mercury lamp. The CCD wavelength was converted to fluorescence wavelength by taking into account the wavelength of the gate pulses. The chirp was removed from the data with the help of the chirp polynomial (Eq. S4) and photometric correction function was applied. Finally, the averaged and corrected spectra were transferred into the transition dipole moment representation and subjected to further analysis.

S1.5 Time-correlated single photon counting (TCSPC)

Fluorescence lifetime measurements were collected using the time-correlated single photon counting (TCSPC) technique, based on the PicoQuant HydraHarp 400 model. The excitation source consisted of a PicoQuant PDL 800-D pulsed diode laser driver with a LDH-PC-375 diode laser emitting at 375 nm. The spectral full-width half maximum (FWHM) of the output beam was approximately 4 nm. Excitation pulses were generated at a repetition rate of 16 MHz for all measurements. The beam was passed through a Berek compensator with variable retardance, which was used as a half-wave plate to control the polarization

of excitation light. Fluorescence monitoring at 520 nm was achieved with the use of a bandpass filter centered at 520 nm with a 6 nm transmission band. To determine the temporal resolution of the experiment, a scattering sample was employed, resulting in an estimated value of approximately 80 ps for the 375 nm excitation (FWHM of the instrument response function).

The experiment involving **NMI**, where the monitoring wavelength was 670 nm (Fig. 5 of the main text), was performed on another setup described in detail previously.[10] Briefly, the experiment was performed on a home-built TCSPC setup using pulsed laser diodes (LDH-PC-400b) for excitation at 395 nm. The beam was passed through a Glan-Taylor polarizer to ensure linearly polarized excitation before reaching the sample. The detector was a photomultiplier tube (PMT, Hamamatsu). The instrument response function (IRF) measured using Ludox in water provided a resolution of approximately 200 ps. Fluorescence was monitored at 670 nm using a bandpass filter centered at 670 nm with a 10 nm transmission band.

All measurements were carried out in a 10 mm quartz cuvette. Fluorescence was collected at the magic angle with respect to the excitation beam in a right-angle geometry, using interference filters and detected by PMT. The excitation power was adjusted below the limit where the count rate was below 1% of the repetition rate of the diode laser. The bin width of traces recorded was 8 ps. All measurements were conducted at room temperature and ambient conditions.

S1.6 Nanosecond transient absorption (ns-TA) spectroscopy

The visible transient absorption (TA) data presented in this work were recorded with a nanosecond TA (ns-TA) setup. A detailed description of the general principle of the ns-TA applying referenced detection with two spectrographs is presented elsewhere.[11] The design of the ns-TA probe beam path is identical to that of the fs-TA presented in ref. 11. The absorbance of the samples at the excitation wavelength was 0.1-0.2 in 1 mm optical path. The samples were measured in 1 mm quartz cuvettes (Starna, model 1GS/Q/1) and bubbled with nitrogen during the experiments. The absorption spectra of all samples showed no sign of degradation after the experiments.

Broadband probing was achieved using white-light pulses generated by focusing the 800 nm pulses of the 5 kHz Ti:Sapphire amplified (Spectra Physics, Solstice Ace) system in a 3 mm CaF_2 plate. The 800 nm pulses were chopped down to 1 kHz repetition rate by mechanical choppers before generation of the white-light pulses. The experimental layout was the same as that described earlier,[12] except that all lenses, after white light generation,

were replaced by spherical mirrors to prevent chromatic aberration.

Excitation scheme for the ns-TA has been described in detail in ref. 13. Excitation was performed at 355 nm using a passively Q-switched, frequency tripled Nd:YAG laser (3, Teem Photonics, Powerchip NanoUV) producing pulses with a 500 Hz repetition rate, approximately 20 μJ energy, and 300 ps duration. This limits the time resolution of the instrument to about 350 ps. However, the early time spectra contain a lot of noise due to stochastic collection of the signal.[13]

The pixel to wavelength conversion was achieved using a standard containing rare earth metals (holmium oxide), which exhibits narrow absorption bands from the UV to the visible spectral region. All transient absorption spectra were corrected for the background signals showing up before time zero (e. g. spontaneous emission).

S2 Ground-state association and deprotonation constants

The derivation of the fit functions for 1:2 host(H)-guest(G) association is heavily adopted from the approach presented in ref. 14. Only the key equations are reproduced here but all intermediate equations are given in refs. 14 and 15.

The two equilibrium reactions and corresponding association constants are:

$$\mathrm{H} + \mathrm{G} \rightleftharpoons \mathrm{HG} \qquad K_1 = \frac{[\mathrm{HG}]}{[\mathrm{H}][\mathrm{G}]} \tag{S5}$$

$$\mathrm{HG} + \mathrm{G} \rightleftharpoons \mathrm{HG_2} \qquad K_2 = \frac{[\mathrm{HG_2}]}{[\mathrm{HG}][\mathrm{G}]} \tag{S6}$$

where K_1 and K_2 are the association constant for the formation of 1:1 and 1:2 complexes, respectively, and [X] corresponds to the concentration of the free host (H), free guest (G), 1:1 complex (HG) or 1:2 complex (HG_2). The total concentration of each species can be derived from the stoichiometry according to:

$$[\mathrm{H}]_0 = [\mathrm{H}] + [\mathrm{HG}] + [\mathrm{HG_2}] \tag{S7}$$

$$[\mathrm{G}]_0 = [\mathrm{G}] + [\mathrm{HG}] + 2[\mathrm{HG_2}] \tag{S8}$$

Using the above relations, expressions for [H], [HG], and [HG_2] can be derived in terms of a single concentration dependent unknown, [G], and the constants, $[H]_0$ K_1 and K_2. Similarly,

$[G]_0$ can be expressed as:

$$[G]_0 = [G] + \frac{K_1[G] + 2K_1K_2[G]^2}{1 + K_1[G] + K_1K_2[G]^2}[H]_0 \tag{S9}$$

The above equation can be rearranged to solve for [G] resulting a cubic equation. The correct root can be estimated analytically (smallest positive real number) or numerically using the Newton-Raphson method, for example. Once [G] has been solved, all the concentrations can be calculated analytically.

With an expression for one of the concentrations in hand, we need to derive a relationship between the absorption and the guest concentration, [G]. We start by expressing the total absorbance as a function of molar fractions, *f*, and assume that the free guest does not absorb at the monitoring wavelength (this can be experimentally achieved by selecting the monitoring wavelength higher than the guest absorption or by subtracting the guest concentration from the sample concentration, we use the latter).

$$A(\lambda) = A_{\mathrm{H}}(\lambda)f_{\mathrm{H}} + A_{\mathrm{HG}}(\lambda)f_{\mathrm{HG}} + A_{\mathrm{HG_2}}(\lambda)f_{\mathrm{HG_2}} \tag{S10}$$

where $A(\lambda)_i$ is the absorption of each species. Using $f_X = [\mathrm{X}]/[\mathrm{H}]_0$ and substituting the concentrations into eq. (S10) we obtain the final expression that is used in the non-linear least squares fitting.

$$A(\lambda) = \frac{A_{\mathrm{H}}(\lambda) + A_{\mathrm{HG}}(\lambda)K_1[\mathrm{G}] + A_{\mathrm{HG_2}}(\lambda)K_1K_2[\mathrm{G}]^2}{1 + K_1[\mathrm{G}] + K_1K_2[\mathrm{G}]^2} \tag{S11}$$

The fitting parameters are the absorptions of the host, $A_{\mathrm{H}}(\lambda)$, 1:1 complex, $A_{\mathrm{HG}}(\lambda)$, and 1:2 complex, $A_{\mathrm{HG_2}}(\lambda)$, corresponding to the total concentration of the host, $[\mathrm{H}]_0$, and the association constants, K_1 and K_2. The total host and guest concentrations are known from the experimental conditions. An experimental titration curve is obtained by plotting the absorption as a function of the total guest concentration. The complete spectra of all the species can be obtained by fitting the absorption spectra over a broad wavelength range.

We have previously demonstrated that weak bases form neutral hydrogen-bonded complexes with the **C_4-dHONI** photoacid. Moreover, the association shows non-cooperative behavior i.e. $K_1 = 4K_2$. This was implicitly assumed in the analysis to reduce the number of variables. Strong bases, on the other hand, will at least partially deprotonate a single hydroxyl group followed by an association of a second base to the remaining free hydroxyl group.[15] Moreover, the association is shows strong negative cooperativity i.e. $K_1 > 4K_2$. Therefore, the first association constant, K_1 represents a deprotonation constant and is ex-

pected to respond strongly to the polarity of the solvent mixture.

Representative absorption spectra of **C_4-dHONI** upon addition of **NMI** and **DBU** in a **PA/BuCN** solvent mixture with $\Delta f = 0.48$ are presented in Figure 1 of main text. The spectra and best-fit results in all mixtures are given in Figures S3 and S4 and the association/deprotonation constants are summarized in Table S2.

At 200 mM concentration of **NMI**, used in the FLUPS measurements, the relative populations of the free photoacid, 1:1 and 1:2 complexes are about <5%, 30% and >65%, respectively. At above 200 mM, changes in the relative fractions within a range of few tens of mM **NMI** are rather small. In any case, the samples used in the time resolved experiments contain a mixture of the different species. Since binding of a single **NMI** does not change the association constant for the second binding event, the pK_a must also remain constant. Therefore, we would anticipate similar ESPT dynamics for the 1:1 and 1:2 complexes. However, ESPT in 1:2 complex could be sligthly faster due to the fact that the deprotonation can be initiated in either of the two -OH groups but it is clear from the data (Section S4 asnd the main text) that even the 1:1 complex undergoes ultrafast ESPT. Therefore we assume that the 1:1 and 1:2 ion pair species behave similarly and are not resolved separately. The remaining fraction (<5%) of the free photoacids gives rise to the long-lived fluorescence signal at the ROH* band region.

Table S2: Ground-state association/deprotonation constants between C_4-dHONI photoacid and organic bases (NMI and DBU) in PA/BuCN mixtures obtained from the global analysis

	with **NMI**		with **DBU**	
Δf	K_1/M^{-1}	K_2/M^{-1}	K_1/M^{-1}	K_2/M^{-1}
0.389	43 ± 4	11 ± 1	2450 ± 90	301 ± 11
0.433	43 ± 4	11 ± 1	2550 ± 80	292 ± 13
0.475	41 ± 4	10 ± 1	2950 ± 60	297 ± 11
0.518	41 ± 4	10 ± 1	5400 ± 70	325 ± 10
0.561	45 ± 4	11 ± 1	74000 ± 3000	1500 ± 44

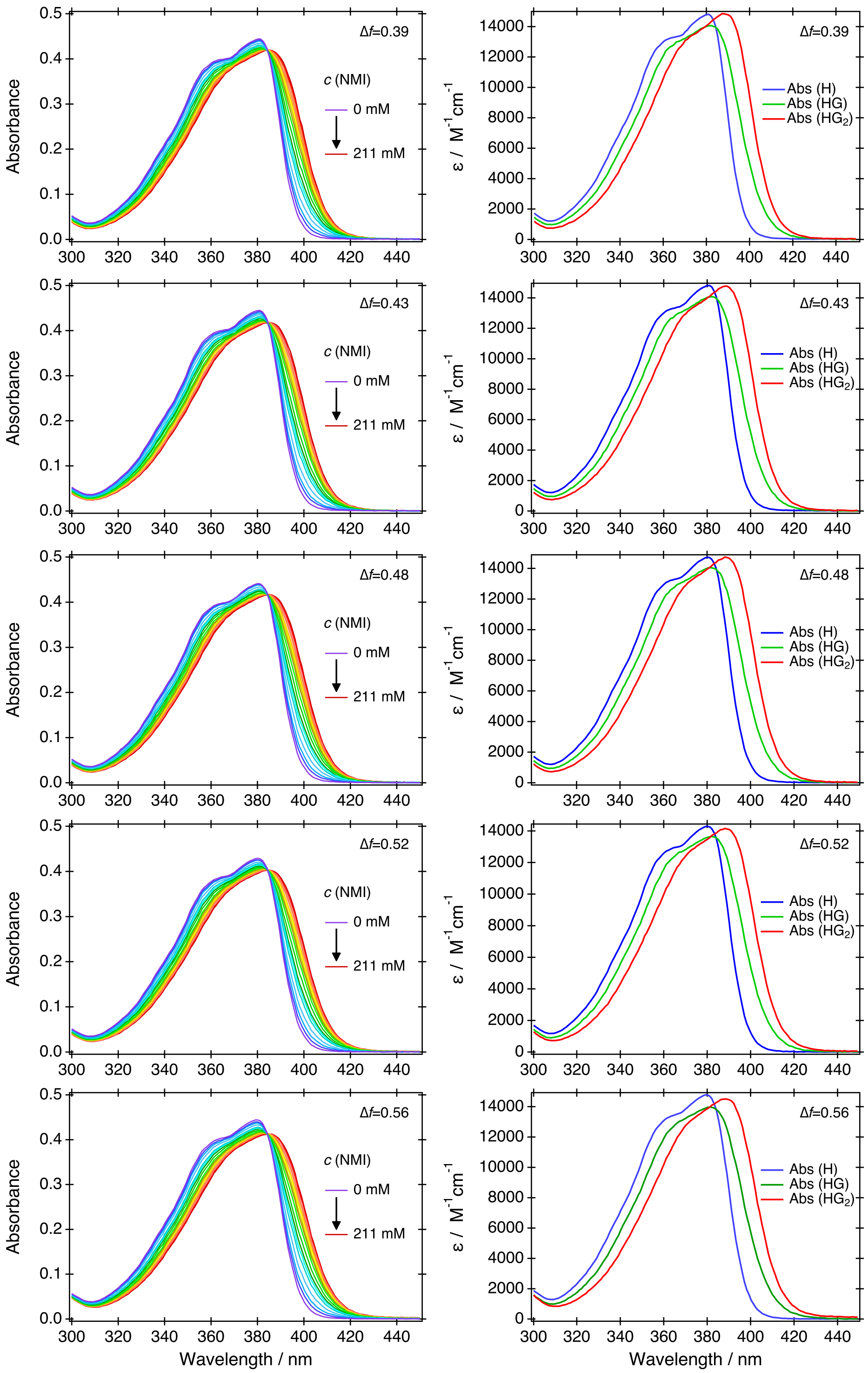

Figure S3: Steady-state absorption spectra of **C_4-dHONI** (c = 30 μM) upon addition of **NMI** in a **PA**/**BuCN** solvent mixture at increasing Δf (top to bottom). The species spectra obtained from global analysis are given in the right panels, where H is **C_4-dHONI** and G is **NMI**.

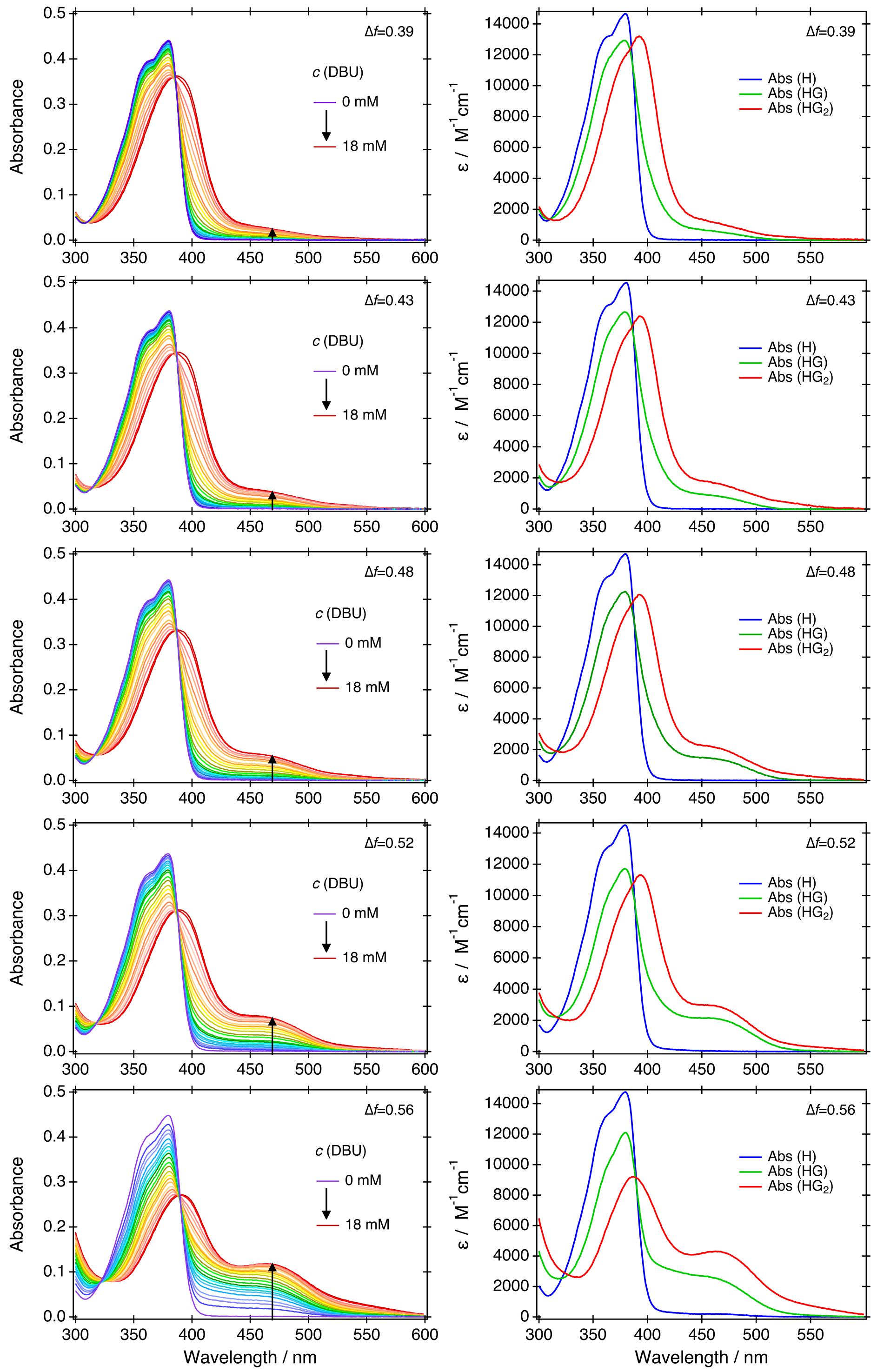

Figure S4: Steady-state absorption spectra of **C$_4$-dHONI** (c = 30 μM) upon addition of **DBU** in a **PA**/**BuCN** solvent mixture at increasing Δf (top to bottom). The species spectra obtained from global analysis are given in the right panels, where H is **C$_4$-dHONI** and G is **DBU**.

S3 Steady-state spectra and band-shape analysis

Steady-state absorption and emission of the free photoacid are presented in Figure S5. The observed shifts can be rationalized based on solvatochromism. The absorption shows very small or negligible shifts probably reflecting the relatively small permanent dipole moment in the ground state. Fluorescence, on the other hand, exhibits a clear positive solvatochromism that can be attributed to the increased permanent dipole moment in the excited state due to the charge-transfer type excitation.[1,15]

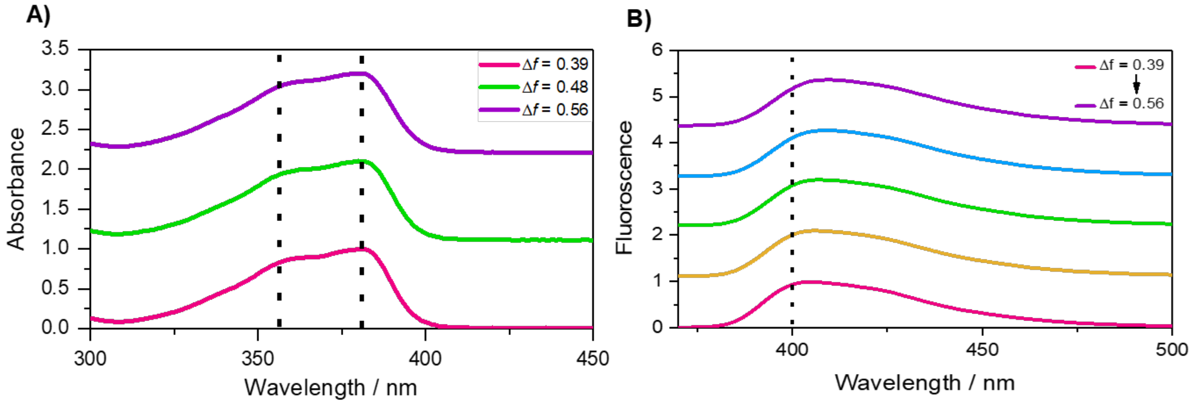

Figure S5: A) Steady-state absorption spectra of the free **C_4-dHONI** in the binary mixtures with Δf of 0.39, 0.48, and 0.56. B) Steady-state emission spectra of the free **C_4-dHONI** in the binary mixtures with Δf of 0.39, 0.43 0.48, 0.52 and 0.56.

Fluorescence spectra of **C_4-dHONI** upon addition of **NMI** in the binary mixtures with Δf of 0.39, 0.48, and 0.56 are presented in Figure S6. The spectra are given in the so-called transition dipole moment representation and have been normalized to the absorbance at the excitation wavelength. Upon increasing concentration of **NMI** the ROH* band centered at $24 \cdot 10^3$ cm^{-1}decreases in intensity and two new low-energy emission bands at about $17.5 \cdot 10^3$ cm^{-1} and $15 \cdot 10^3$ cm^{-1}appear. The low-energy bands are attributed to the CIP* and SSIP/FIP* forms. The quenching of the ROH* band is comparable in all solvents but the enhancement of the SSIP/FIP* band is significantly stronger at $\Delta f = 0.56$. Moreover, the CIP* is clearly resolved at $\Delta f = 0.39$ and 0.48 but overlaps signigicantly with the SSIP/FIP* band at $\Delta f = 0.56$. Lastly, it should be noted, that a slight quenching of the total fluorescence occurs at the highest **NMI** concentrations at $\Delta f = 0.39$ and 0.48.

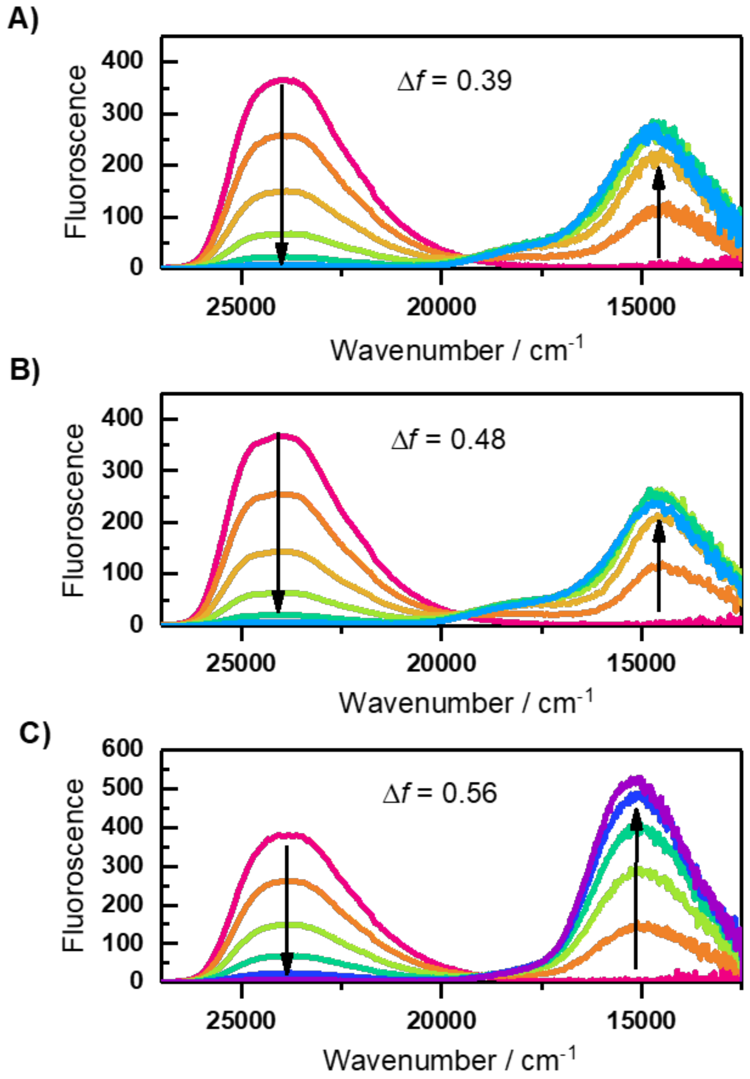

Figure S6: Steady-state fluorescence spectra of **C_4-dHONI** ($c = 12\mu$M) upon addition of **NMI** in a **PA**/**BuCN** solvent mixture with Δf of A) 0.39, B) 0.48 and C) 0.56. Concentration of **NMI** was varied from 0 to 160 mM. The spectra are given in the transition dipole moment representation and have been normalized by absorbance at the excitation wavelength.

S3.1 Band-shape analysis of the steady-state fluorescence spectra

Band-shape analysis of the steady-state fluorescence spectra of **C_4-dHONI** in the presence of **NMI** was performed to separate the contributions from the CIP* and SSIP*/FIP* forms. In our previous publication the SSIP* and FIP* bands were resolved as separate contributions.[15] However, in that study Gaussian band-shape functions were used to decompose the spectra in the wavenumber domain ($F(\tilde{\nu})$). It is clear now that the spectra do not generally have a Gaussian band shape but are better reproduced by the log-normal band-shape function that accounts for the broadening towards the low-energy side.[16] Thus, the previous decomposition appears as an oversimplification. As seen in the steady-state emission spectra (Fig. S6), the SSIP* and FIP* forms overlap too much to be resolved separately and thus their contribution is indicated as SSIP*/FIP* and modeled with a single log-normal function.

The band-shape analysis involved the following steps:

a) Emission spectra of **C_4-dHONI** in the presence of large excess of **NMI** (255 mM) were acquired upon 360 nm excitation alongside with the corresponding absorption spectra.

b) Emission spectra were corrected for both the spectral sensitivity of the instrument and the primary inner filter effect.[17] After these corrections, the intensities are directly proportional to the absorbance at the excitation wavelength. To account for the slight differences in absorbance between different samples, the emission spectra were normalized by the corresponding absorbance values. After this, the intensities are comparable across different samples.

d) The emission spectra were finally transferred into the so-called transition dipole moment representation and analyzed with a sum of two log-normal functions, one corresponding to the CIP* band and the other to the SSIP/FIP* band. The spectra were analyzed from $21 \cdot 10^3$ cm^{-1} down to $12 \cdot 10^3$ cm^{-1} to focus solely on the contributions from the deprotonated species. The spectral parameters of the free ROH* were determined from the sample in the absence of **NMI**.

The equation for the log-normal function is given in the main text (Eqs. 3 and 4). The area (A) and FWHM of the log-normal function have the following analytical forms:

$$A = I_0 \frac{\Delta x}{2} \exp\left[\frac{b^2}{4 \ln 2}\right] \sqrt{\frac{\pi}{\ln 2}}, \tag{S12}$$

$$\text{FWHM} = \Delta x \frac{\sinh(b)}{b}. \tag{S13}$$

In the above equations, I_0 is the peak intensity, b the asymmetry parameter, $\tilde{v}_0$ the peak frequency, Δx the width parameter and A is the area.

Spectral fits in the presence of **NMI** in all five solvent systems are presented in Figures

S7–S11. All spectral parameters are summarized in Tables S3 and S4. The peak frequencies and FWHM of all species are additionally summarized in Table 1 of the main text. The relative areas of the CIP* and SSIP/FIP* bands as a function of Δf are graphically presented in Fig. 2C of the main text, whereas the peak frequencies are presented in Fig. S12.

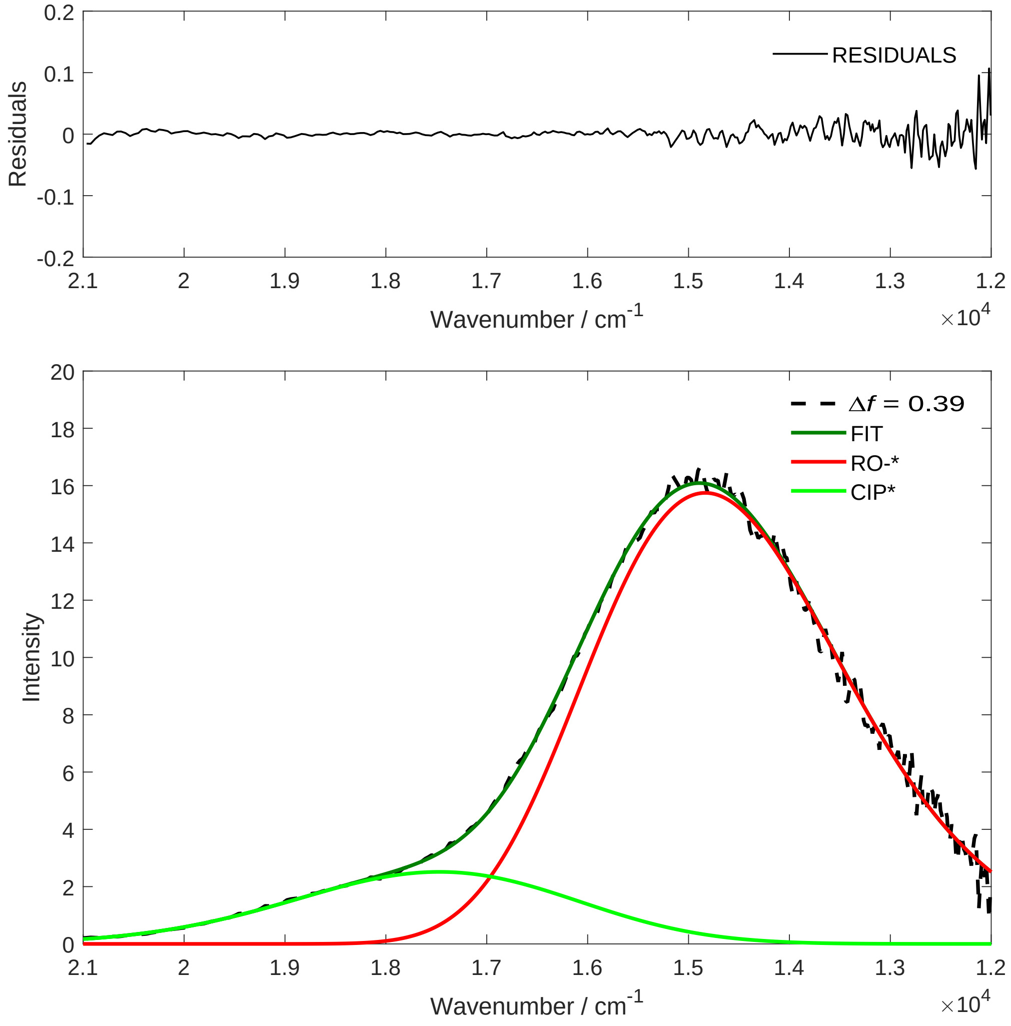

Figure S7: Steady-state emission spectra of **C_4-dHONI** ($c = 13$ μM) in the presence of of **NMI** ($c = 255$ mM) in **PA** ($\Delta f = 0.39$) together with the fit with a sum of two log-normal functions. The residual is given in the top panel.

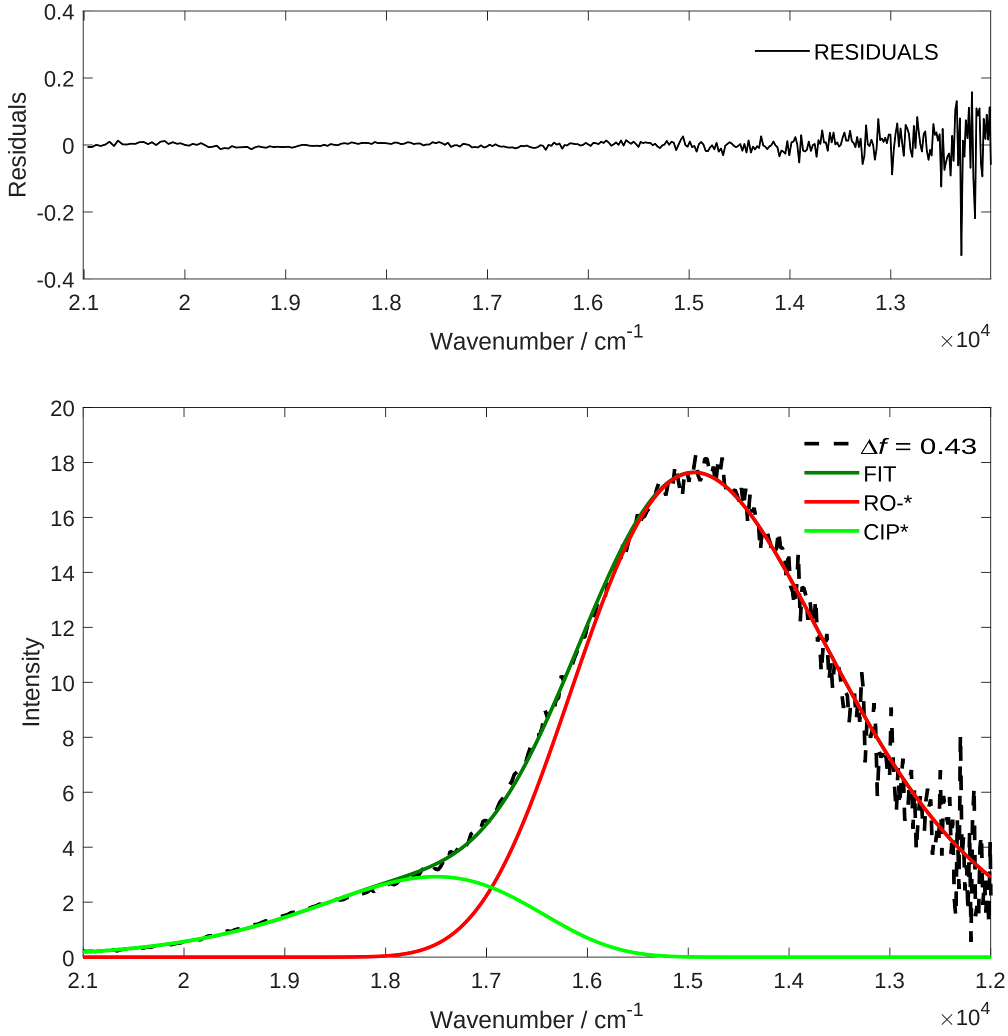

Figure S8: Steady-state emission spectra of **C_4-dHONI** (c = 13 μM) in the presence of of **NMI** (c = 255 mM) in a **PA/BuCN** mixture with $\Delta f = 0.43$ together with the fit with a sum of two log-normal functions. The residual is given in the top panel.

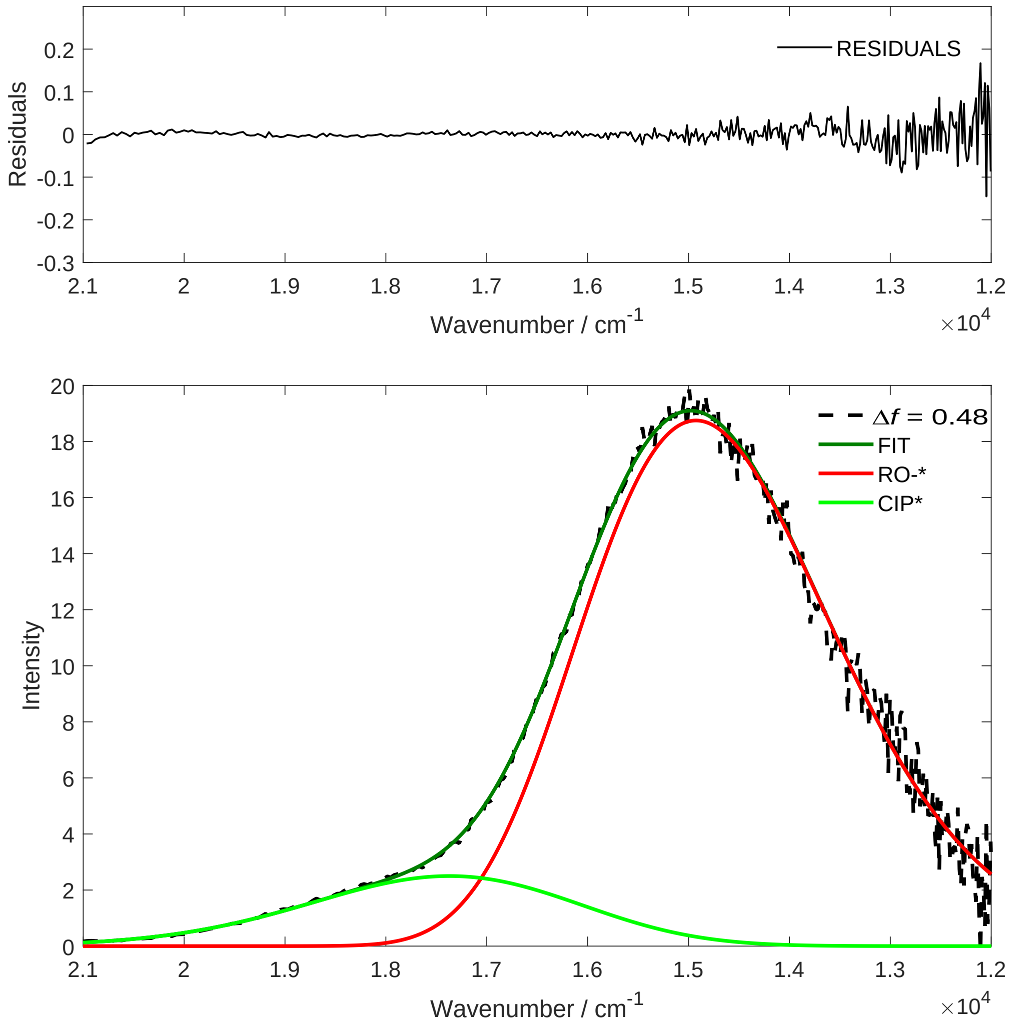

Figure S9: Steady-state emission spectra of $\mathbf{C_4}$**-dHONI** (c = 13 μM) in the presence of of **NMI** ($c = 255$ mM) in a **PA**/**BuCN** mixture with $\Delta f = 0.48$ together with the fit with a sum of two log-normal functions. The residual is given in the top panel.

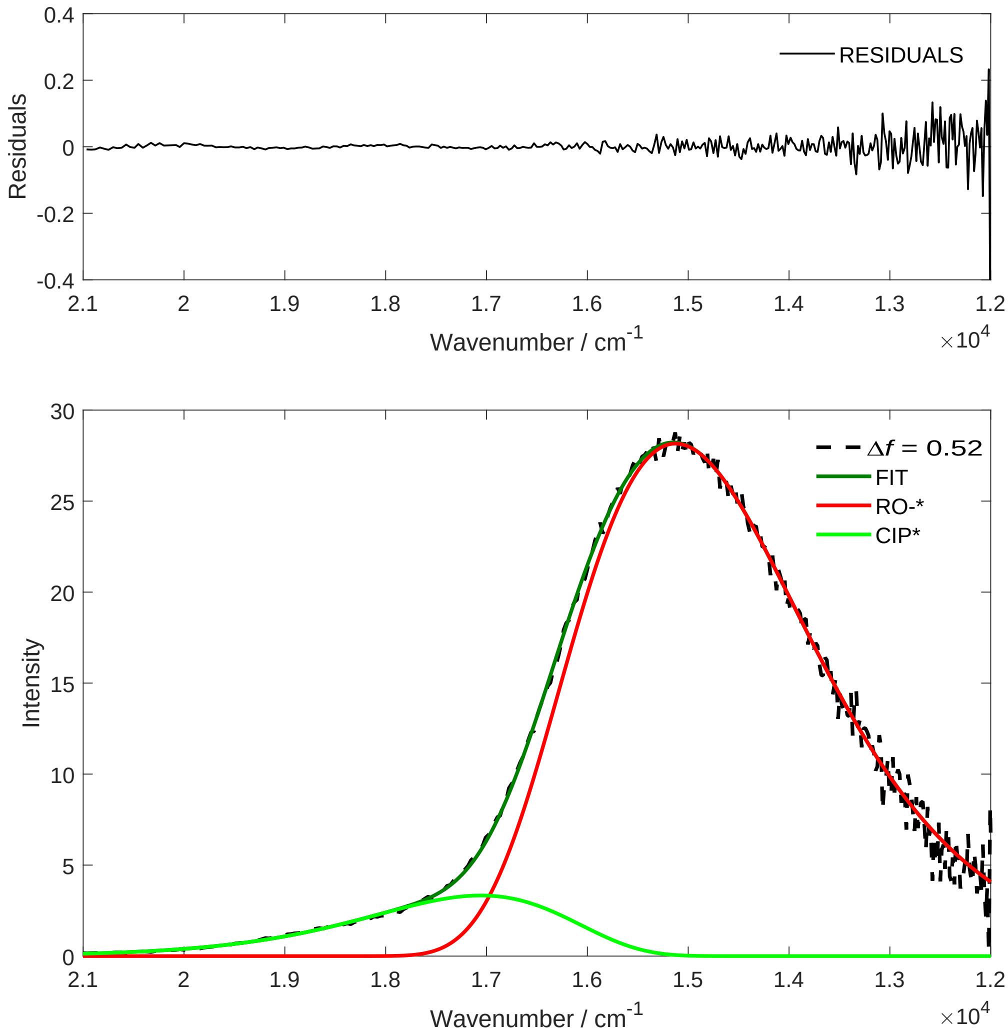

Figure S10: Steady-state emission spectra of **C_4-dHONI** ($c = 13$ μM) in the presence of of **NMI** ($c = 255$ mM) in a **PA**/**BuCN** mixture with $\Delta f = 0.52$ together with the fit with a sum of two log-normal functions. The residual is given in the top panel.

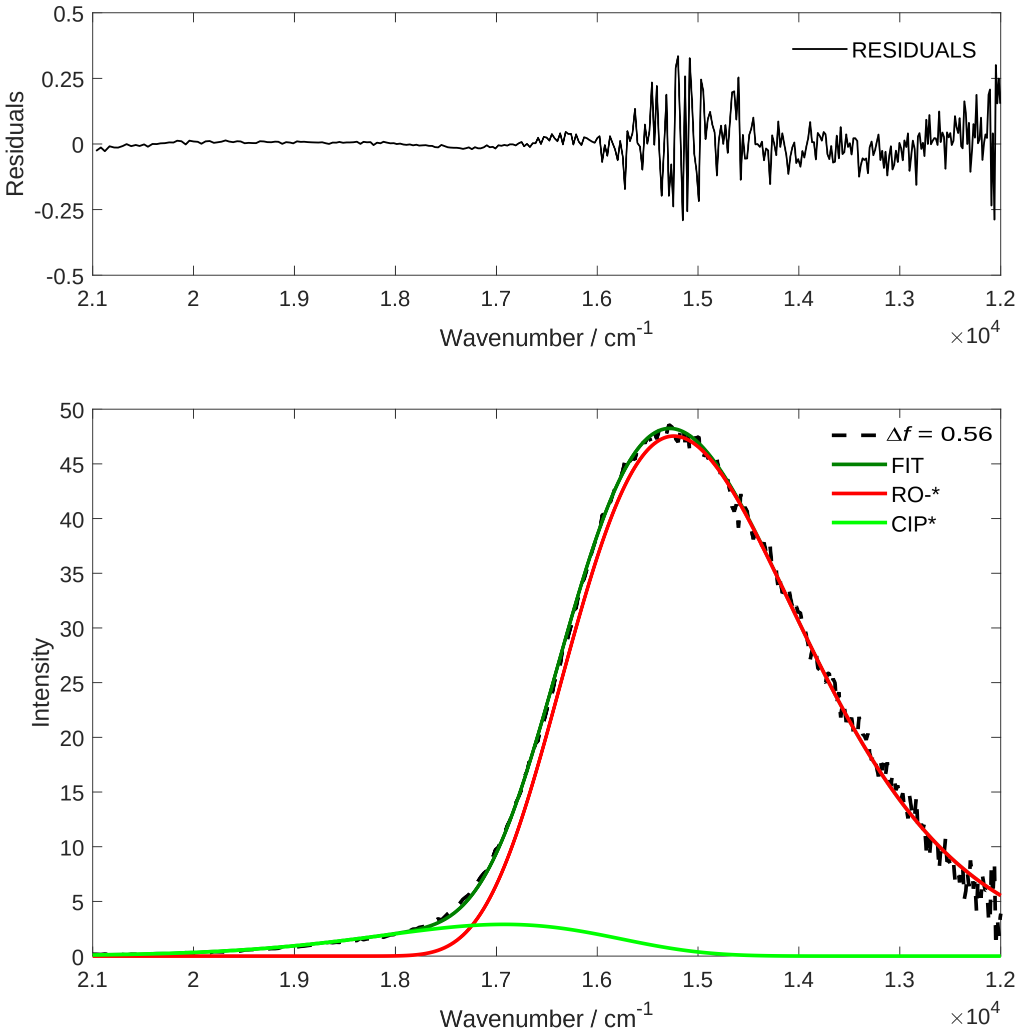

Figure S11: Steady-state emission spectra of **C_4-dHONI** (c = 13 μM) in the presence of of **NMI** (c = 255 mM) in **BuCN** (Δf = 0.56) together with the fit with a sum of two log-normal functions. The residual is given in the top panel.

Table S3: Fluorescence band-shape parameters of the CIP* band in solvent mixtures

Δf	I_0	Δx	b	$\tilde{v}_0$	A
0.39	2.51	3294	0.08	17480	3.22×10^3
0.43	2.93	2560	0.3	17490	3.16×10^3
0.48	2.5	3120	0.1	17370	3.06×10^3
0.52	3.3	2400	0.36	17060	3.53×10^3
0.56	2.9	2760	0.25	16920	3.30×10^3

Table S4: Fluorescence band-shape parameters of the SSIP/FIP* band in solvent mixtures

Δf	I_0	Δx	b	$\tilde{v}_0$	A
0.39	15.7	2986	-0.18	14834	1.88×10^4
0.43	17.6	2949	-0.25	14930	2.14×10^4
0.48	18.7	2916	-0.19	14927	2.19×10^4
0.52	28.1	2786	-0.33	15126	3.39×10^4
0.56	47.5	2713	-0.33	15241	5.56×10^4

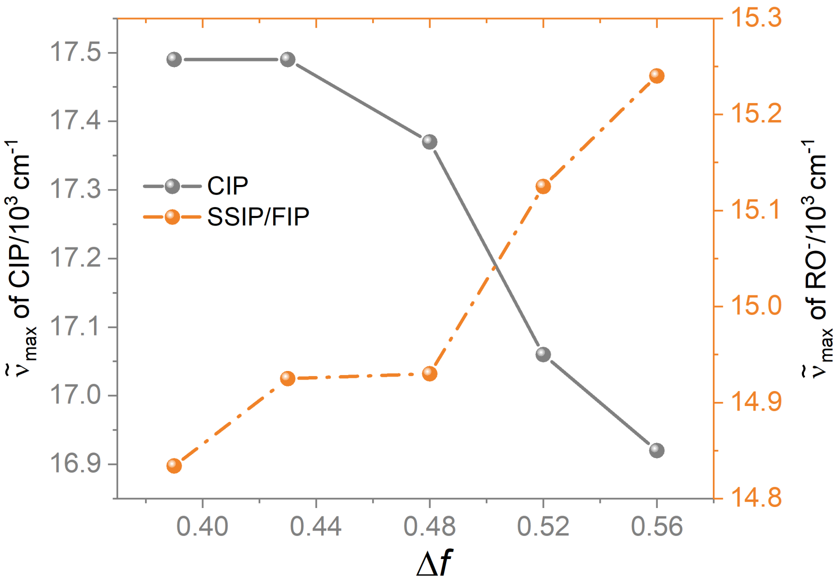

Figure S12: The peak frequencies of the CIP* and SSIP/FIP* bands as a function of Δf.

S4 Broadband fluorescence up-conversion spectra

To obtain the final fluorescence spectra of **C_4-dHONI** in the presence of **NMI**, we measured the fluorescence count distributions at three crystal angles as described in section S1.4. This was necessary to achieve adequate S/N over the full detection range of about 400 nm ($14 \cdot 10^3$ cm^{-1}). This was particularly important for the red part of the spectrum above 650 nm because the phase matching efficiency decreases and the TDM representation amplifies the noise in this part of the spectrum. To further improve the S/N, more scans were averaged in the crystal angle particularly optimized for the red part. A total of 15 scans with 1 s integration time at each time step were averaged at the crystal angles optimized for the blue and green parts of the spectrum whereas 30 scans were averaged for the red part of the spectrum. Some of the scans were discarded from the final data due to scattering of the pump and/or the third harmonic of the gate pulses. The final count distribution was obtained as the sum of the individual measurements measured at the three crystal angles. The sample was continuously stirred by bubbling with nitrogen during the experiments. Degradation of the sample was monitored by measuring the absorption before and after the experiments. The change in absorbance at the maximum was less than 10% for all samples.

The same procedure was used for the solvent blank and reference measurements but with fewer scans. The chirp due to GVD was found to be independent of the crystal angle and was therefore collected at a single angle optimized for the **BBOT** reference dye. Full procedure for generating the photometric correction function from the measurements performed at the three crystal angles is described elsewhere.[9]

The concentration of the samples were adjusted to achieve $A = 0.3 - 0.4$ in 1 mm cuvette at the excitation wavelength of 399 nm. The samples were prepared by evaporating the required amount of the stock solution and redissolving it in 2 mL of the solvent of choice. Finally, neat **NMI** was added to the solution.

The final fluorescence spectra of **C_4-dHONI** ($c = 350\mu$M) in the presence of **NMI** ($c = 200\mu$M) in **PA**, one mixture with $\Delta f = 0.48$ denoted as **MIX**, and **BuCN** are presented in Figure S12 as 3D-plots. Representative spectra at selected time points in all three solvent systems are presented in Figure 3 of the main text.

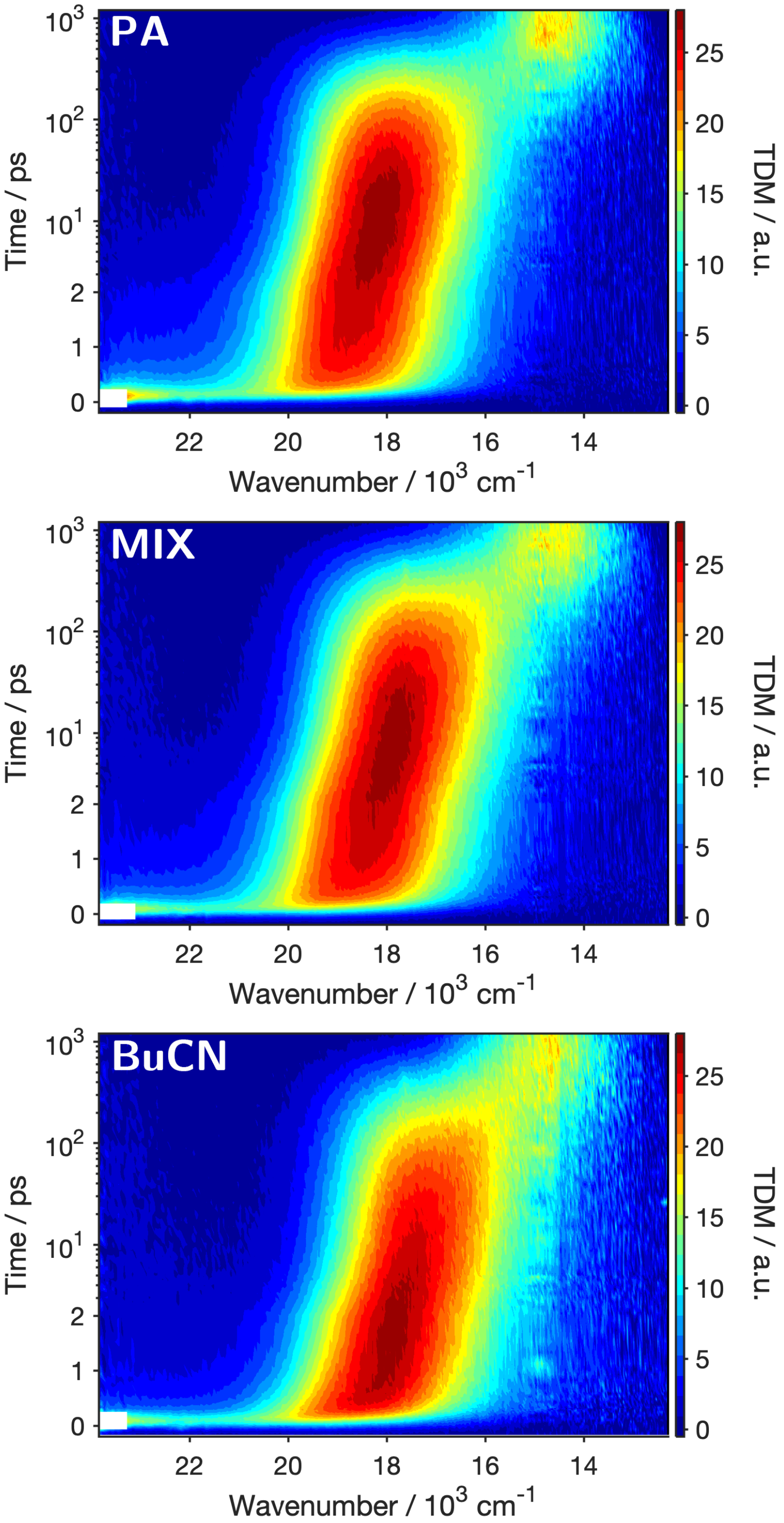

Figure S13: Time-resolved fluorescence spectra of **C_4-dHONI** (350 μM) in the presence of **NMI** (200 mM) in (top) **PA**, (middle) **MIX** with Δf = 0.48, and (bottom) **BuCN**. Excitation wavelength was 399 nm.

S4.1 Band-shape analysis of the FLUPS spectra

In our previous studies,[16,18] we introduced a time-dependent band-shape analysis for resolving the contributions from spectrally overlapping fluorescent species. In such analysis, each of the emitting species is modeled as a single time-dependent log-normal function (Eq. 3 in the main text). The advantage of this analysis is that the solvation dynamics can be explicitly taken into account as time-dependent red shifts of the log-normal functions whereas the population dynamics can be accessed independently from the band areas. Furthermore, a kinetic reaction scheme, such as that presented in Scheme 1 of the main text, can be imposed on population dynamics. However, the first-order kinetic schemes are unable to account for the multi-exponential dynamics that limit the result from the nonequilibrium dynamics.[16] Therefore, full global analysis based on a kinetic scheme was not attempted here.

Instead, we tried to resolve the contributions from the individual species by simple spectral analysis where the spectra at each time step are analyzed with a sum of three log-normal functions to obtain the spectral parameters. These spectral parameters were then analyzed with multi-exponential functions to obtain the kinetic parameters. However, the multi-exponential kinetics were not connected to a particular kinetic scheme. The main limitations of the band-shape analysis arise from the following facts. First of all, contribution from the ROH* form and rise of the CIP* form is poorly resolved due to the ultrafast ESPT. This particularly influences determination of the ultrafast rise components. Secondly, the residual Raman signal overlaps with the ROH* band. However, the large uncertainty in the intensity reduces the weight of these regions in the fit. Third, the CIP* and SSIP*/FIP* bands overlap to large extent and their contributions could not be fully resolved without significant constraints on the fitting parameters. Despite these challenges, the band-shape analysis allowed us to resolve the most important population dynamics and capture the solvent relaxation of the CIP* form.

The results of the band-shape analysis in **PA**, **MIX**, and **BuCN** are presented in Figure S14, where the markers represent the spectral parameters obtained from the analysis of the individual spectra at each time step and the lines represent the multi-exponential functions. The following constraints were imposed on the parameters. The relaxed band frequencies and spectral widths of the CIP* and SSIP*/FIP* bands were constraint close ($\pm 5\%$) to the values obtained from the steady-state spectra. The asymmetry parameter, b, was assumed to remain negative for all species. The band frequency of the SSIP*/FIP* band was assumed to remain constant, whereas one- or two-exponential relaxation was assumed for the ROH* and CIP* bands. The decays were modeled as two- to three-exponential functions that include the rising components.

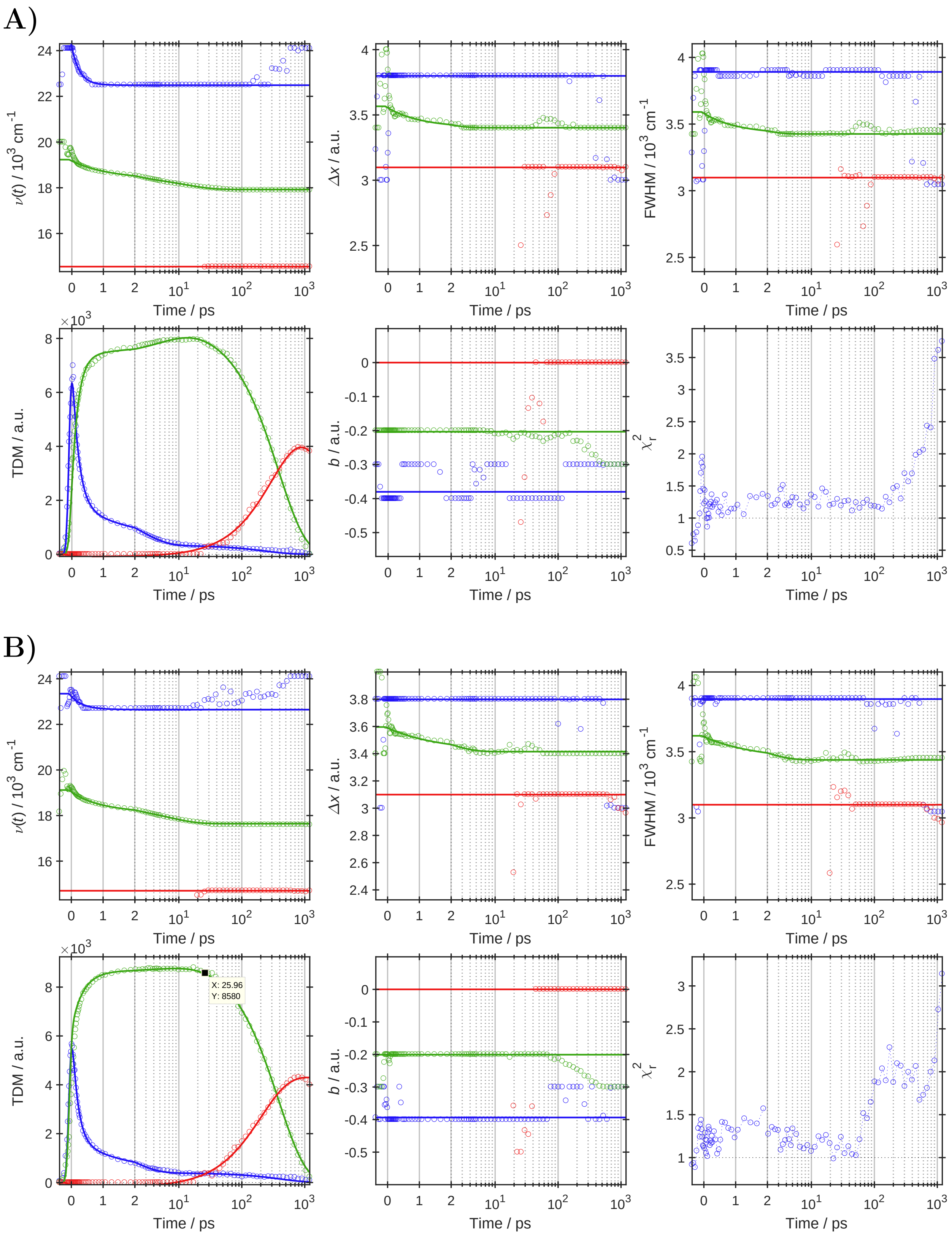

(figure continues on the next page)

C)

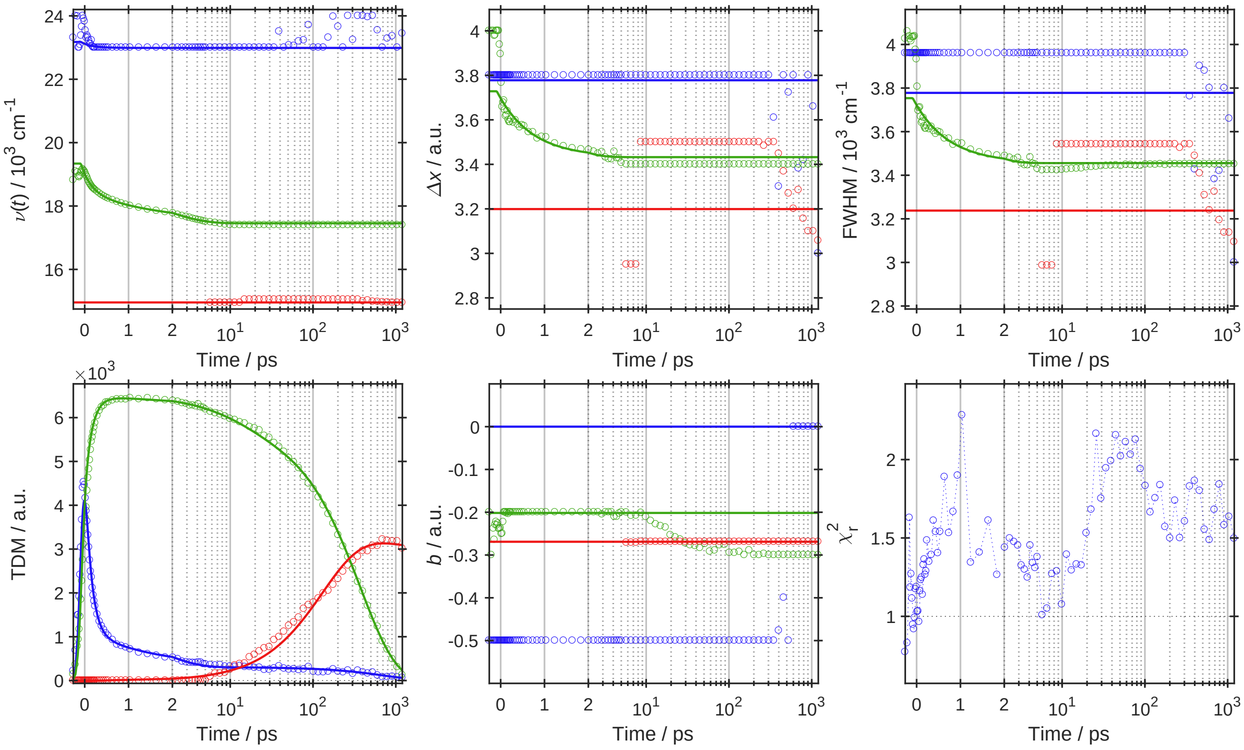

Figure S14: Evolution of the log-normal band-shape parameters in A) **PA**, B) **MIX** and C) **BuCN**. The parameters are (top left) the band frequencies, (top middle) the width parameter, (top right) FWHM, (bottom left) area, and (bottom middle) asymmetry. χ_r^2 values of the spectral fits at each time step are given in bottom right panels. The markers represent the spectral parameters at each time step and the lines represent the multi-exponential functions.

The spectral parameters support the qualitative observations discussed in the main text. We focus our discussion here on the band frequencies and the areas. Due to the ultrafast decay, the band frequency of the ROH* band is poorly resolved and was limited to about 22–23 $\cdot 10^3$ cm^{-1} to prevent it from merging with the CIP* band. On the other hand, the CIP* band is relatively well resolved in all solvents. This band undergoes a significant dynamic Stokes shifts during the first 10–30 ps. The time scale depends on the solvent, being significantly faster in **BuCN** in line with our previous reports on the solvent relaxation in these solvents.[2] Solvent relaxation in completed in about 10 ps in **BuCN** whereas it extends up to 30 ps in **PA**. The relaxed CIP* band frequency ($\tilde{\nu}_\infty$) is smaller in **BuCN** (17.5 $\cdot 10^3$ cm^{-1}) than in **PA** (17.9 $\cdot 10^3$ cm^{-1}) due to the larger solvation energy. Expectedly, the relaxed band frequency in **MIX** (17.6 $\cdot 10^3$ cm^{-1}) is in between the values obtained in the neat solvents. These frequencies are slightly smaller than those determined from the steady-state spectra (Table 1 in the main text) but the uncertainties in the latter are relatively large.

The dynamic Stokes shifts were modeled with two-exponential functions to extract the time constants, amplitudes and total shifts. The parameters are summarized in Table S5 together with previously reported values for mean solvation times ($< \tau_{\mathrm{solv}} >$).[2]

Table S5: Spectral and kinetic parameters of the time-dependent Stokes shift of the CIP* band. The spectral parameters are given in 10^3 cm^{-1}, amplitudes in % and time constants in ps

Solvent	$\Delta\tilde{\nu}_{\mathrm{tot}}$	$\tilde{\nu}_{\infty}$	α_1	τ_1	α_2	τ_2	$< \tau_{\mathrm{ave}} >^a$	$< \tau_{\mathrm{solv}} >^b$
**PA**	1.3	17.9	0.56	1.1	0.44	12.7	6.2	2.3±0.2
**MIX**	1.5	17.6	0.50	0.8	0.50	7.2	4.0	3.9±0.3
**BuCN**	1.9	17.5	0.46	0.2	0.54	1.8	1.1	0.8±0.1

[a] The average is calculated from the lifetimes and amplitudes as $< \tau_{\mathrm{ave}} >= \Sigma\alpha_i\tau_i$. [b] Mean solvation times from ref. 2.

The magnitude of the total shift increases upon increasing polarity but difference between **PA** and **MIX** is smaller than the difference between **MIX** and **BuCN**. The time constants become significantly smaller in more polar environment. The average time constants in **MIX** and **BuCN** agree well with the mean solvation times determined from dynamics Stokes shift of standard push-pull dyes.[2] However, in **PA** the average time constant is significantly slower than the mean solvation time. This might be due to poor modeling of the CIP* band at early times due to its low intensity resulting in overestimation of the initial band position. Another plausible reason is that the presence of large concentration of the more polar **NMI** results in preferential solvation in nonpolar **PA** increasing the magnitude of the total shift. Secondly, such preferential solvation often occurs on slower time scale compared to normal solvation dynamics of neat solvents. However, this was not investigated further.

It is evident from the band areas that the decay of the ROH* band and rise of the CIP* band are significantly faster in **BuCN** than in **PA**. Again, the time scale in **MIX** is in between those of the neat solvents. The CIP* reaches its maximum intensity at about 1–2 ps in **BuCN** whereas it takes nearly 10 ps to reach the maximum in **PA**. A direct comparison of decay times of the CIP* band and the rise of the SSIP*/FIP* band is more challenging due to the delayed onset of the CIP* band in **PA**. Secondly, the CIP* and SSIP*/FIP* bands are poorly separated in the spectral fits between 10 and 100 ps due to the significant spectral overlap and low intensity of the SSIP*/FIP* band. This is manifested as overestimation of the SSIP*/FIP* band area and underestimation of the CIP* band area in this time range. Mixing of the two bands is evident from the disagreement between the spectral fits (markers) and the exponential functions (lines) in Fig. S14. To partially eliminate the mixing, the spectra were modeled with only two log-normal functions up to 25, 20 and 5 ps in **PA**, **MIX**, and **BuCN**, respectively. After 100–200 ps, the SSIP*/FIP* band is better resolved from the

CIP* band and the exponential fits agree well with the results of the spectral fits. In all solvents, the final band area of the SSIP*/FIP* is about 50% from the CIP* band and reaches its maximum at about 700–800 ps. Due to the TDM representation, the band area is directly proportional to the yield of the SSIP*/FIP* form and appears to be relatively independent of the polarity.

In all solvents, a long-lived minor decay component of the ROH* band with a lifetime of several hundreds of ps appears in the spectral fits that can be attributed to a small fraction of the free photoacid present at 200 mM **NMI** concentration. However, its magnitude is likely overestimated. Due to the poor resolution of the ROH* band, the width (Δx and b) of the log-normal function becomes very large, as evident from the FWHM value, and the band extends towards lower frequencies over the CIP* band region. It was difficult to impose meaningful parameter constraints to prevent the broadening of the ROH* band. Nevertheless, the amplitude is very small for all samples (3–4%).

The band areas of the ROH* and CIP* bands were modeled with two- or three-exponential functions whereas the SSIP*/FIP* band was modeled with a two-exponential function. The exponential functions were convolved with a Gaussian-simulated IRF to account for the finite duration of the excitation pulse. The same IRF parameters were used for all three decays of the same sample but some variation was allowed between the different samples. The FWHM of the IRF, resulting from the fits, were in the range of 150–170 fs. The two fast decay components of the ROH* band and rise components of the CIP* band reflect the ESPT

Table S6: Lifetimes and amplitudes of the band areas extracted from the multi-exponential fits of the spectral parameters. The long-lived decay components of the ROH* and SSIP*/FIP* bands are omitted for clarity. The lifetimes are given in ps and the amplitudes in %

	ROH* decay					CIP* rise				
**Solvent**	α_1	τ_1	α_2	τ_2	$< \tau_{ave} >^a$	α_1	τ_1	α_2	τ_2	$< \tau_{ave} >^a$
**PA**	0.84	0.21	0.16	2.4	0.57	0.87	0.20	0.13	6.6	1.05
**MIX**	0.83	0.18	0.17	1.7	0.45	0.83	0.36	0.17	9.2	1.81
**BuCN**	0.88	0.14	0.12	1.6	0.30	1.00	0.16	– [b]	– [b]	0.16

	CIP* decay		SSIP*/FIP* rise
**Solvent**	$\alpha_{rise}/\alpha_{decay}$ [c]	τ_1	τ_1
**PA**	0.98	380	410
**MIX**	0.37	380	210
**BuCN**	0.49	370	130

[a] The average is calculated from the lifetimes and amplitudes as $< \tau_{ave} >= \Sigma \alpha_i \tau_i$. [b] Only a single rise component was resolved in **BuCN**. [c] Ratio of the total amplitudes of the rising and decaying components.

process. The long-lived component of the the ROH* band accounts for the residual tail and was omitted from Table S6 for clarity (not discussed further). The slower decay component of the CIP* band and rise component of the SSIP*/FIP* band reflect the dissociation of the CIP* form. The decay of the SSIP*/FIP* band occurs on a longer time scale beyond the time scale of the FLUPS instrument and the lifetime was also omitted from Table S6. The lifetime was resolved in the TCSPC experiments (Section S5 and the discussion in the main text). Lifetimes and amplitudes in all solvents are summarized in Table S6.

Initial ESPT is clearly accelerated upon increasing polarity. Both fast decay components of the ROH* band are fastest in **BuCN** and the amplitude of the fastest component slightly increases. The average decay time, calculated as the amplitude weighted average of the two decay components, is reduced from 570 fs in **PA** to 300 fs in **BuCN**. A more thorough discussion on the decay parameters is provided in the main text.

The rise of the CIP* band should correlate with the decay of the ROH* band but the trend is much less clear across the different solvents. A fast component in the order of few hundreds of fs is resolved in all solvents but the slower rise components, resolved in **PA** and **MIX**, are significantly longer than the longer decay component of the ROH* band. We note that a much better agreement is obtained by limiting the analysis to the first 30 ps where only ROH* and CIP* forms are present (results not shown). It should be also noted that less than 50% of the total CIP* rise is resolved in **MIX** and **BuCN** in the analysis presented in Figure S14. Therefore, we rely on the decay of the ROH* band in estimating the average ESPT time. The decay of the CIP* band, on the other, is better resolved an appears relatively independent of the solvent with a value of about 380 ps. Due to the mixing of the CIP* and SSIP*/FIP* bands, the decay of teh CIP* form was independently analyzed from the actual decay trace averaged close to the band maximum. The wavelength region for the averaged signal is presented in Fig. 3 by the green vertical fill. This region does not significantly overlap with the SSIP*/FIP* band and allows for determination of the CIP* decays. The decays were analyzed by three-exponential functions and the lifetimes and amplitudes are presented in Table S7.

Table S7: Lifetimes and amplitudes of the exponential fits to the averaged CIP* decay traces from the FLUPS spectra in different solvents. The CIP* band at around 560 ± 5 nm. Negative amplitudes indicate a rising component

**Solvent**	α_1	τ_1 / fs	α_2	τ_2 / ps	α_3	τ_3 / ps
**PA**	–0.30	590	–0.17	4.7	1.00	400
**MIX**	–0.27	240	–0.25	1.3	1.00	410
**BuCN**	–0.35	350	0.15	17.7	0.86	410

The fast rise and decay components of the CIP* band reflect both the ESPT and solvation dynamics that cannot be separated. The only relevant lifetime is the last decay component (τ_3) that reflects the decay of the CIP* form. Similarly to the band-shape analysis, the decay time is independent of the solvent with a value of about 410 ps, very close to the value obtained from the band-shape analysis (380 ps). Due to uncertainty caused by the spectral mixing with the SSIP*/FIP* band, only the results of the exponential analysis of the direct decay traces are discussed in the main text.

Lastly, the rise of the SSIP*/FIP* band agrees with the decay of the CIP* band in **PA** but is significantly faster in **MIX** and **BuCN**. This results from the larger spectral mixing between the CIP* and SSIP*/FIP* bands in these solvents.

It should be noted that the exponential analysis presented here is applied to the spectral parameters obtained from the log-normal band-shape modeling. Thus, a meaningful statistical weighting and error analysis was challenging especially in cases were the parameters had to be constraint to certain values. The direct decay traces of the CIP* band, presented in Figure 4B of the main text, were analyzed by a weighted fit that takes into account statistically meaningful uncertainties of the original data. We also attempted to analyze the direct decay of the ROH* band (data not presented) but this resulted in much shorter (sub-IRF) lifetimes with large uncertainties in the amplitudes. This is most likely due to the solvent relaxation and residual Raman signal, not accounted for in the analysis of the direct decay. Nevertheless, both analyses yield a similar qualitative picture of the influence of the dielectric constant on the individual steps.

S5 Nanosecond fluorescence decays (TCSPC)

Nanosecond fluorescence decays were investigated by TCSPC method. The decay traces were analyzed by multi-exponential functions convolved with an experimentally measured IRF. The decays were monitored at different monitoring wavelengths by the use of interference filters. The decays of **C_4-dHONI** in the presence of **NMI** in different **PA**/**BuCN** mixtures monitored at 670 nm were collected on a different TCSPC setup as described in section S1.5. The experimental decays, fits and weighted residuals for the 670 nm monitoring wavelength are presented in Figure S15. The lifetimes and amplitudes resulting from the exponential analysis are given Table S8. The values are additionally presented in Table 2 and discussed in the main text.

The decays of **C_4-dHONI** in the presence of **NMI** were additionally monitored at 520 nm. The experimental decays, fits and weighted residuals are presented in Figure S16. The lifetimes and amplitudes, resulting from the exponential analysis, are given in Table S9. The lifetime of the dominant decay component upon 520 nm monitoring is about 350 ps, in good agreement with the lifetime determined from the FLUPS measurements. The dominant decay component is accompanied by two slower decay components with ns lifetimes. In **PA**, the lifetime is close to the decay time attributed to the SSIP* form and the amplitude of the longest-lived component is negligible. Upon increasing polarity, the amplitude of the longest-lived component increases and te lifetime of the intermediate component is reduced. This is likely due to a poor resolution of the two components with relatively small amplitudes. Therefore, determination of the lifetime of the SSIP* form is more reliable upon 670 nm monitoring, where this decay component is the dominant component.

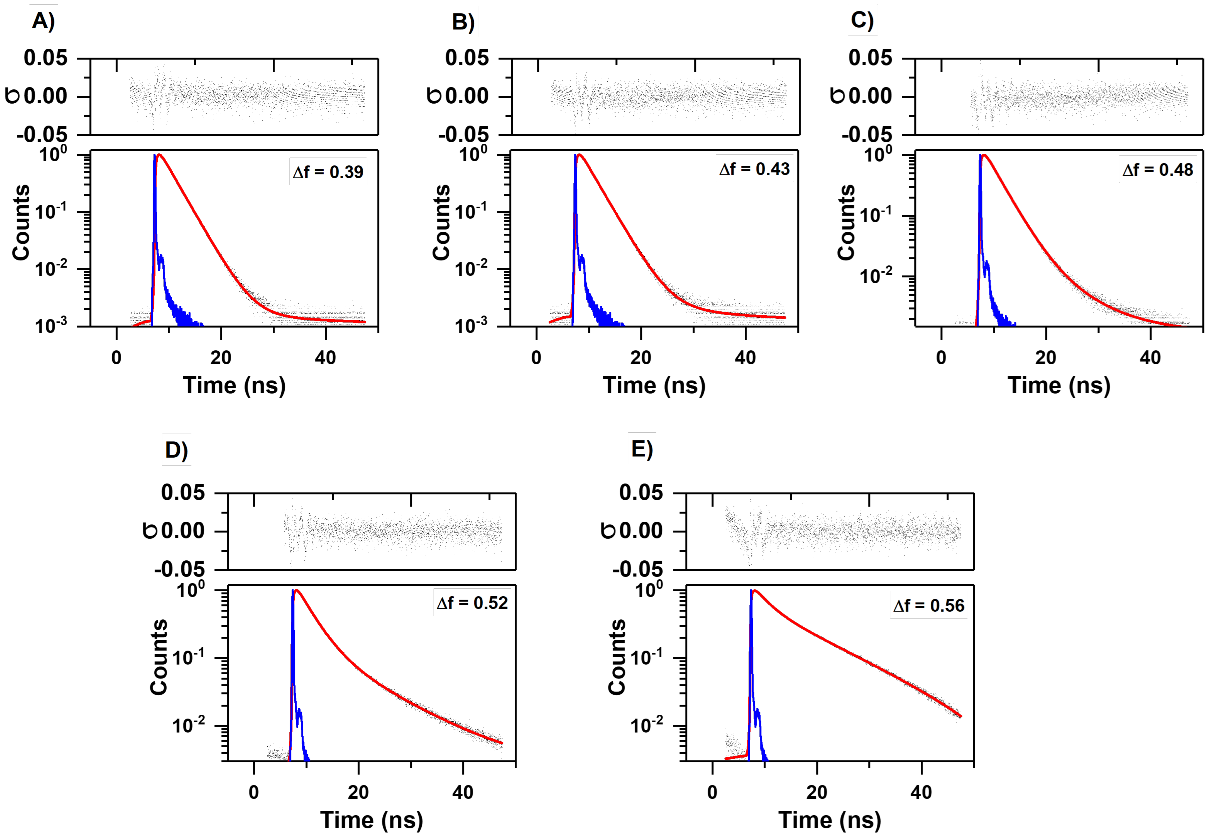

Figure S15: Fluorescence decay traces of **C_4-dHONI** (15 μM) in the presence of 220 mM **NMI**, monitored at 670 nm upon 395 nm excitation together with the exponential fits and weighted residuals. Δf values of each mixture are given in the insets.

Table S8: Lifetimes and χ^2 values for C_4-dHONI in the presence of NMI in different PA/BuCN mixtures, measured using TCSPC, upon excitation at 395 nm and monitoring at 670 nm. The values are obtained from the convolution of the IRF with a multi-exponential function

Δf	a_1	τ_1 / ns	a_2	τ_2 / ns	a_3	τ_3 / ns	χ^2
0.39	Rise	0.37	1	2.7	-	-	1.12
0.43	Rise	0.36	1	2.7	-	-	1.13
0.48	Rise	0.38	0.98	2.8	0.02	7.0	1.18
0.52	Rise	0.35	0.79	2.8	0.21	10.1	1.17
0.56	Rise	0.39	0.53	2.7	0.47	12.1	1.31

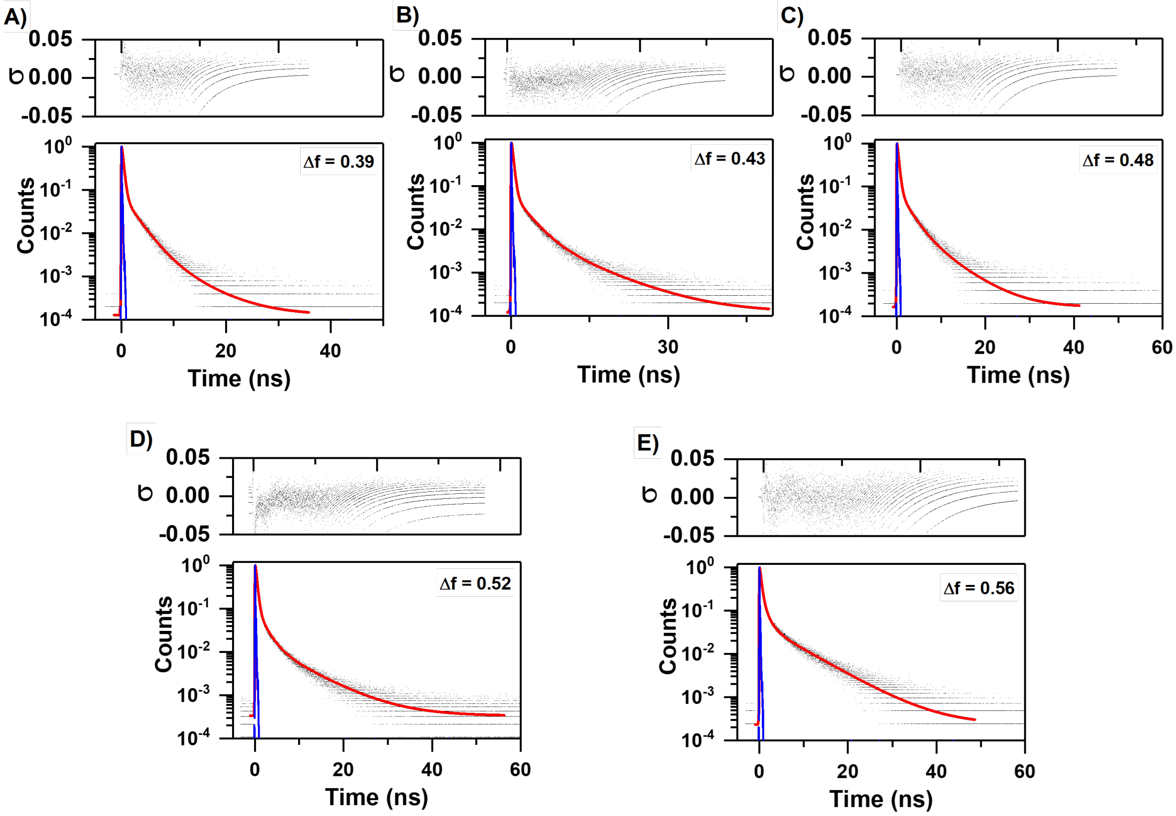

Figure S16: Fluorescence decay traces of **C_4-dHONI** (13 μM) in the presence of 300 mM **NMI**, monitored at 520 nm upon 375 nm excitation together with the exponential fits and weighted residuals. Δf values of each mixture are given in the insets.

Table S9: Lifetimes and χ^2 values for C_4-dHONI in the presence of NMI in different PA/BuCN mixtures, measured using TCSPC, upon excitation at 375 nm and monitoring at 520 nm. The values are obtained from the convolution of the IRF with a multi-exponential function

Δf	a_1	τ_1 / ns	a_2	τ_2 / ns	a_3	τ_3 / ns	χ^2
0.39	0.96	0.34	0.04	2.6	0.003	6.1	1.15
0.43	0.95	0.32	0.04	2.5	0.01	8.3	1.20
0.48	0.95	0.35	0.04	2.1	0.01	5.8	1.16
0.52	0.92	0.34	0.06	1.8	0.02	7.6	1.20
0.56	0.83	0.37	0.14	1.2	0.03	7.4	1.10

S6 Nanosecond transient absorption (ns-TA)

Formation of the fully dissociated ground-state ions was investigated by the ns-TA method. Transient absoprtion spectra of **C_4-dHONI** in the presence of **NMI** were measured in all solvent mixtures. Representative time-resolved spectra in **PA** and **BuCN** are presented in Figure S17 as 3D-plots. The first 20 ns, displaying a strong excited-state absorption (ESA) signal above 400 nm and ground-state bleach (GSB) below 400 nm, is left out for clarity. Thus, the signals in Fig. S17 mostly arise from the ground-state species.

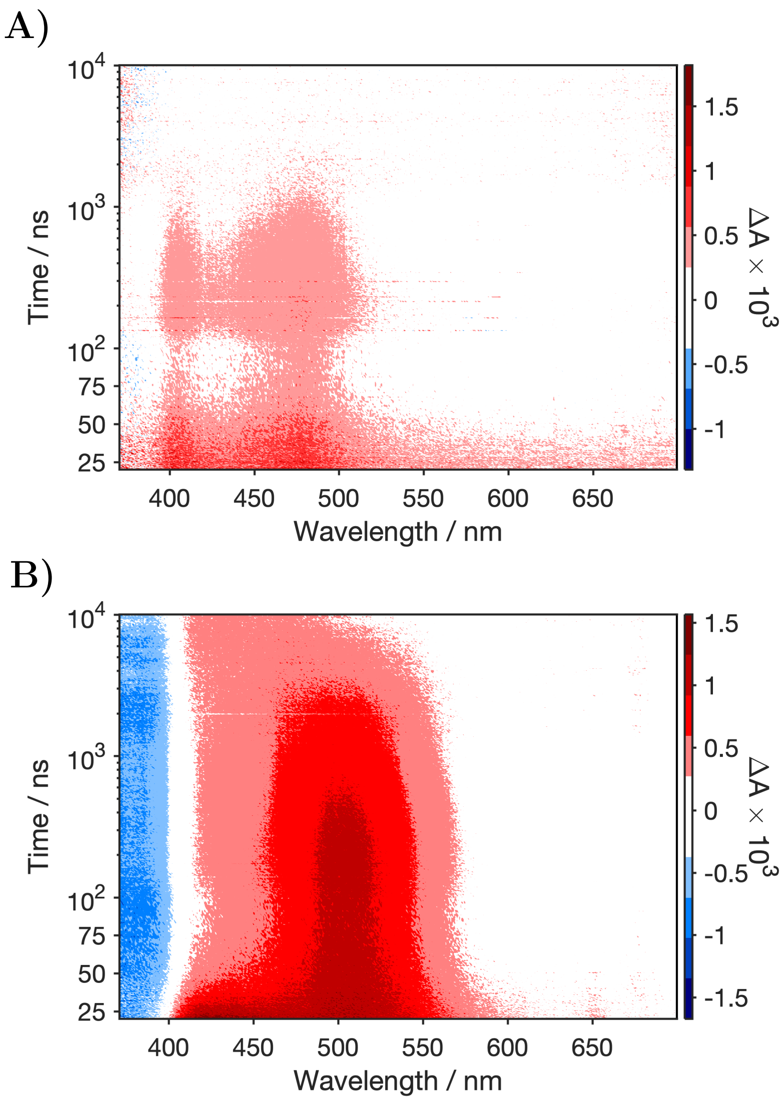

Figure S17: Nanosecond transient absorption spectra of **C_4-dHONI** ($c = 175\ \mu$M) in the presence of **NMI** ($c = 200$ mM) in A) **PA** and B) **BuCN** upon 355 nm excitation. The first 20 ns, displaying a strong excited-state absorption (ESA) and ground-state bleach (GSB) signals, is left out for clarity.

The behavior is distinctly different in the two neat solvents. First of all, the excited-

state lifetime is significantly longer in **BuCN** and the tail of the strong ESA is detectable up few tens of ns whereas in **PA** the excited-state decay is nearly complete at about 25 ns, in agreement with the TCSPC measurements. After excited-state decay, a long-lived component extending up to several μs can be detected in both solvents. However, the signal is much weaker and has a different spectral shape in **PA**. In **PA**, the long-lived component exhibits two distinct absorption maxima at around 400 and 475 nm whereas in **BuCN** a single dominant absorption maximum at around 500 nm and a persistent GSB signal below 400 nm are observed (Fig. S17). Moreover, the decay of this component is much slower in **BuCN** extending up to several μs.

The time-resolved ns-TA spectra were analyzed by means of conventional global analysis with two- or three-exponential kinetic models.[19] The resulting spectra are given as Evolution Associated Difference Spectra (EADS) that reflect the species spectra from a sequential model without any back reactions or loss channels. Thus, the EADS should be considered as an empirical representation of the spectral evolution and the associated time scales characterized by the lifetimes τ_i. EADS and associated lifetimes in all solvents are presented in Figure S18 and Table S10.

Before discussing the results we note that the decrease in the intensity between 50 and 150 ns, clearly detectable in **PA**, is due to a thermal shock induced by the excitation pulse. Its amplitude is significantly smaller in **BuCN** due to lower excitation power used for this sample. At this excitation power, the signal of the long-lived species in **PA** and the mixtures was extremely weak and therefore, ca. two times higher excitation power was used. This also results in two times higher amplitude of the initial ESA signal. The thermal shock does not significantly influence the early and the late kinetics and is only visible in the 50–150 ns time range. Representative time traces together with fits and residuals are presented in Fig. S19A. The poor fit quality from 0 to 30 ns is mostly due to the excited-state dynamics that are represented by a single decay component in the fit model, contrary to the multi-exponential decay. However, the ns excited-state dynamics are accurately captured in the TCSPC experiments (cf. Section S5). The thermal shock is clearly visible in the residuals at around 50–150 ns after which the residuals remain relatively flat. Therefore, the thermal shock has no influence on the interpretation of the results.

Overall spectral and kinetic behavior in solvents with $\Delta f \leq 0.48$ is qualitatively very similar. Initial ESPT is too fast to be resolved within the time-resolution and therefore, the excited-state processes are captured by a single ns-component. This component can be attributed to the excited deprotonated form, namely SSIP*, as evidenced by the stimulated emission observed above 650 nm. SSIP* exhibits a strong ESA band with a maximum at around 420 nm, in agreement with previous reports.[18,20] In addition, a relatively GSB

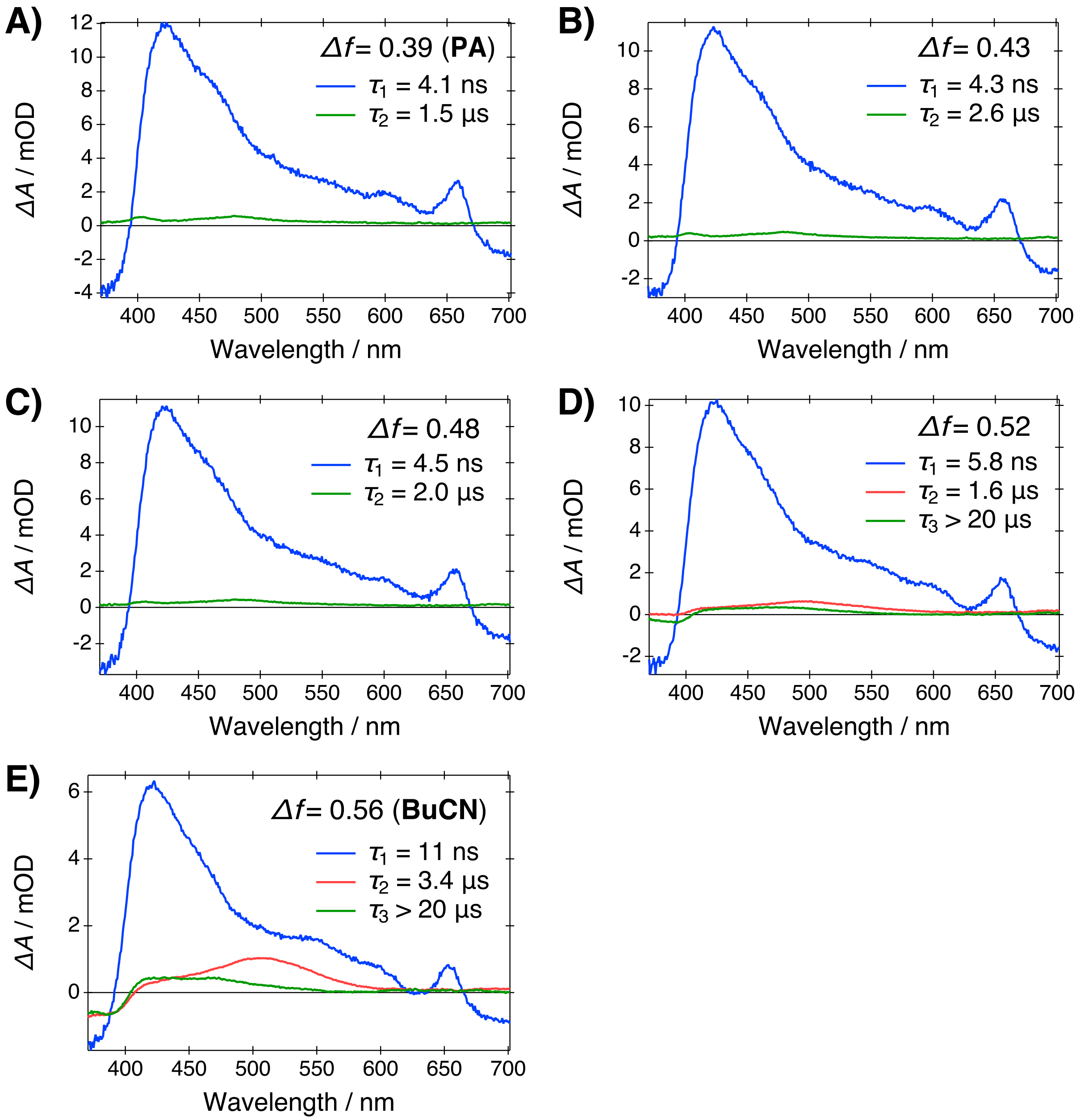

Figure S18: Evolution Associated Difference Spectra resulting from the global analysis of the ns-TA spectra of **C_4-dHONI** (c = 175 μM) in the presence of **NMI** (c = 200 mM). Δf values of the solvents and associated lifetimes are given in the inset.

signal is observed below 400 nm that recovers concomitantly with the disappearance of the strong ESA signal (Fig. S19A). The lifetime is relatively close the 3 ns lifetime resolved in the TCSPC experiments and is relatively independent of the solvent. The excited-state component is followed by a weak long-lived component with lifetime of about 2 μs. EADS corresponding to this component in different solvents are overlaid in Fig. S19C. The spectral

signature of this component with distinct absorption maxima at around 400 nm and 475 nm is nearly identical in solvents with $\Delta f \leq 0.48$. Moreover, the relative amplitudes of the component are almost the same. Interestingly, this component does not exhibit a detectable GSB signal that is most likely masked by the induced absorption. In any case, the overall intensity of this component is very small.

The origin of this component is discussed more thoroughly in the main text where it is attributed to the radical anion of the 1,8-naphthalimide photoacid generated via electron transfer from **NMI**. The nearly identical kinetics, spectral shapes and amplitudes of the EADS in $\Delta f \leq 0.48$ demonstrate that the slow excited-state processed remains unchanged in this polarity range. The only difference is the slight acceleration of the initial ESPT observed in the FLUPS experiments.

At $\Delta f > 0.48$, another intermediate component with an absorption maximum at 500–510 nm appears. This component is attributed to the fully dissociated ground-state anion. The spectral signature of the ground-state anion of **dHONI**-photoacid was already reported in previous study in **MeCN** and **PhCN** using both transient UV-vis and IR-spectroscopies.[20] The ground-state anion was generated via ESPT to an organic base, similarly to the present study, but photoacid was part of a bistable rotaxane. Nevertheless, the spectral position of the anion is comparable to that reported previously and indicates that the anion is not hydrogen bonded to protonated base. Hydrogen bonding interaction results in a significant blue shift of the spectrum. This also explains why the absorption maximum of the ground-state anion in the steady-state spectra measured in the presence of **DBU** appears at shorter wavelength compared to the transient spectrum (Fig. S4). Furthermore, the lack of detectable GSB signal in the transient spectrum of the deprotonated ground-state acid is explained by the significant overlap with the relatively strong $S_2 \leftarrow S_0$ transition observed in the steady-state spectra. It should be noted that the negative signal below 410 nm in the EADS2 (red in Fig. S18E) originates from the bleach of the much longer-lived EADS3 (green in Fig. S18E).

In the present solvent mixtures, both the amplitude and lifetime of the ground-state anion signal depend strongly on the solvent, both increasing upon increasing polarity. A significant population of the ground-state anions is produced only at the highest polarity, although it is also detectable in the mixture with $\Delta f = 0.52$. This observation clearly demonstrates that a long-lived population of the ground-state ions is produced selectively after full dissociation into FIP* in the excited state. In this regard, the fluorescence spectra may yield erroneous conclusions since the SSIP* and FIP* forms appear spectrally very similar. However, the former does not produce long-lived ground-state ions due to fast geminate recombination. The geminate recombination in this case is accelerated by Coulomb interactions that also

Table S10: Summary of the lifetimes obtained from the global analysis of the ns-TA data in all solvents. The first component, τ_1, in all cases corresponds to the excited-state decay. The longest lifetime (τ_2 at $\Delta f \leq 0.48$ and τ_3 at $\Delta f > 0.48$) corresponds to the radical anion whereas τ_2 at $\Delta f > 0.48$ corresponds to the deprotonated ground-state anion

Δf	τ_1 / ns	τ_2 / μs	τ_3 / μs
0.39	4.1	1.5	-
0.43	4.3	2.6	-
0.48	4.5	2.0	-
0.52	5.8	1.6	>20
0.56	11	3.4	>20

prevents the full dissociation in the excited state.

The differences in the kinetic behavior become more apparent in the time traces of the ns-TA data. Averaged signals at around the GSB (370–380 nm) and at around the absorption maximum of the ground-state deprotonated form are presented in Fig. S19A and B, respectively. Due to the differences in the excitation power, the traces have been scaled to have the same initial intensity close to time zero. Again, the dip due to the thermal shock is clearly visible at 50–150 ns. The decay traces in solvents with $\Delta f \leq 0.48$ are virtually identical. Above this value, the amplitudes of the long-lived components starts to increase. The amplitude follows a similar trend in polarity as the sudden increase in the intensity of the steady-state fluorescence attributed to the SSIP*/FIP* as well as the increase in deprotonation constants determined in the presence of **DBU**. This demonstrates that all of these observations originate from the same physical phenomenon that is the full dissociation of the deprotonated acid and the protonated base, either in the ground or excited state.

Overlay of the EADS of the longest-lived components is presented in Fig. S19B. The spectra in solvents with $\Delta f \leq 0.48$ feature two distinct absorption maxima at around 400 nm and 475 nm, as already discussed above. The absorption maxima at $\Delta f > 0.48$ appear slightly blue shifted from the 475 nm and the short-wavelength region is distorted due to the ground-state bleach. Despite the slight differences, we believe that the origin of this component is identical in all solvent systems.

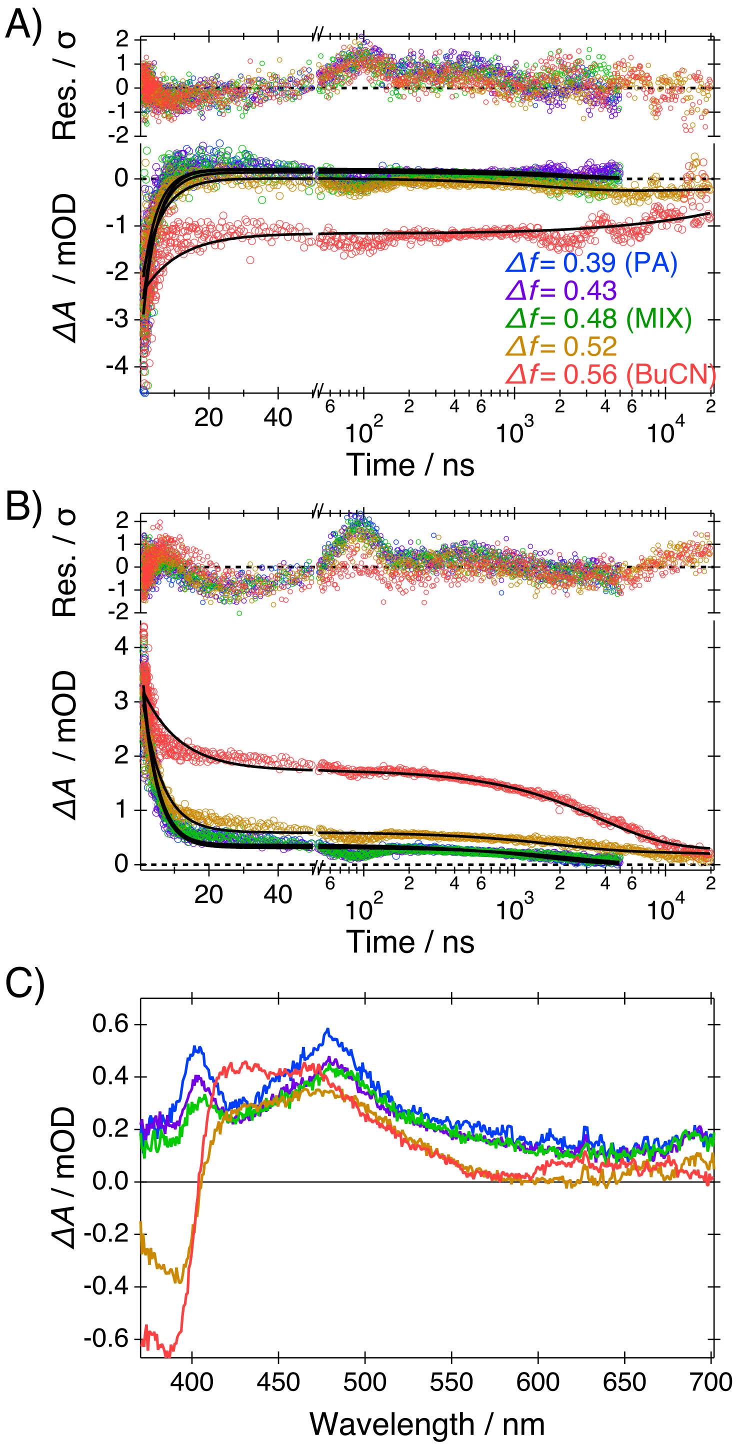

Figure S19: Time traces, fits and residuals of the ns-TA data averaged A) 370–380 nm and B) at 500–510 nm in all solvents. The fits represent the results of the global analysis presented in Fig. S18. The residuals are given in the units of standard deviations. The signal decrease at around 50–150 ns is due to a thermal shock caused by the excitation pulse. C) Overlay of the Evolution Associated Difference Spectra of the longest-lived components (green component in Fig. S18) in all solvent mixtures.

References

(1) Kumpulainen, T.; Bakker, B. H.; Hilbers, M.; Brouwer, A. M. Synthesis and Spectroscopic Characterization of 1,8-Naphthalimide Derived "Super" Photoacids. *J. Phys. Chem. B* **2015**, *119*, 2515–2524.

(2) Verma, P.; Rosspeintner, A.; Kumpulainen, T. Propyl acetate/butyronitrile mixture is ideally suited for investigating the effect of dielectric stabilization on (photo)chemical reactions. *RSC Adv.* **2020**, *10*, 23682–23689.

(3) Reichardt, C. Solvatochromic Dyes as Solvent Polarity Indicators. *Chemical Reviews* **1994**, *94*, 2319–2358.

(4) Marcus, Y. *The Properties of Solvents*, 1st ed.; Wiley: Chichester, 1998.

(5) Gardecki, J. A.; Maroncelli, M. Set of Secondary Emission Standards for Calibration of the Spectral Responsivity in Emission Spectroscopy. *Appl. Spectrosc.* **1998**, *52*, 1179–1189.

(6) Zhao, L.; Luis Pérez Lustres, J.; Farztdinov, V.; Ernsting, N. P. Femtosecond fluorescence spectroscopy by upconversion with tilted gate pulses. *Phys. Chem. Chem. Phys.* **2005**, *7*, 1716–1725.

(7) Zhang, X.-X.; Würth, C.; Zhao, L.; Resch-Genger, U.; Ernsting, N. P.; Sajadi, M. Femtosecond Broadband Fluorescence Upconversion Spectroscopy: Improved Setup and Photometric Correction. *Rev. Sci. Instrum.* **2011**, *82*, 063108.

(8) Gerecke, M.; Bierhance, G.; Gutmann, M.; Ernsting, N. P.; Rosspeintner, A. Femtosecond Broadband Fluorescence Upconversion Spectroscopy: Spectral Coverage Versus Efficiency. *Rev. Sci. Instrum.* **2016**, *87*, 053115.

(9) Kapitzke, M.; Palato, S.; Raj, A.; Kumpulainen, T.; Stähler, J. Enhancing Spectral Coverage, Efficiency, and Photometric Accuracy in Ultrafast Fluorescence Upconversion Spectroscopy. *ChemPhotoChem* **2025**, e202400398.

(10) Fürstenberg, A.; Vauthey, E. Excited-state dynamics of the fluorescent probe Lucifer Yellow in liquid solutions and in heterogeneous media. *Photochemical & Photobiological Sciences* **2005**, *4*, 260–267.

(11) Lang, B. Photometrics of ultrafast and fast broadband electronic transient absorption spectroscopy: State of the art. *Rev. Sci. Instrum.* **2018**, *89*, 093112.

(12) Banerji, N.; Duvanel, G.; Perez-Velasco, A.; Maity, S.; Sakai, N.; Matile, S.; Vauthey, E. Excited-State Dynamics of Hybrid Multichromophoric Systems: Toward an Excitation Wavelength Control of the Charge Separation Pathways. *J. Phys. Chem. A* **2009**, *113*, 8202—-8212.

(13) Lang, B.; Mosquera-Vàzquez, S.; Lovy, D.; Sherin, P.; Markovic, V.; Vauthey, E. Broadband ultraviolet-visible transient absorption spectroscopy in the nanosecond to microsecond time domain with sub-nanosecond time resolution. *Rev. Sci. Instrum.* **2013**, *84*, 073107.

(14) Hargrove, A. E.; Zhong, Z.; Sessler, J. L.; Anslyn, E. V. Algorithms for the Determination of Binding Constants and Enantiomeric Excess in Complex Host:Guest Equilibria Using Optical Measurements. *New J. Chem.* **2010**, *34*, 348–354.

(15) Kumpulainen, T.; Bakker, B. H.; Brouwer, A. M. Complexes of a Naphthalimide Photoacid with Organic Bases, and Their Excited-State Dynamics in Polar Aprotic Organic Solvents. *Phys. Chem. Chem. Phys.* **2015**, *17*, 20715–20724.

(16) Verma, P.; Rosspeintner, A.; Dereka, B.; Vauthey, E.; Kumpulainen, T. Broadband fluorescence reveals mechanistic differences in excited-state proton transfer to protic and aprotic solvents. *Chem. Sci.* **2020**, *11*, 7963–7971.

(17) Kasparek, A.; Smyk, B. A new approach to the old problem: Inner filter effect type I and II in fluorescence. *Spectrochim. Acta, Part A.* **2018**, *198*, 297–303.

(18) Kumpulainen, T.; Rosspeintner, A.; Dereka, B.; Vauthey, E. Influence of Solvent Relaxation on Ultrafast Excited-State Proton Transfer to Solvent. *J. Phys. Chem. Lett.* **2017**, *8*, 4516–4521.

(19) van Stokkum, I. H. M.; Larsen, D. S.; van Grondelle, R. Global and Target Analysis of Time-Resolved Spectra. *Biochim. Biophys. Acta, Bioenerg.* **2004**, *1657*, 82–104.

(20) Kumpulainen, T.; Panman, M. R.; Bakker, B. H.; Hilbers, M.; Woutersen, S.; Brouwer, A. M. Accelerating the Shuttling in Hydrogen-Bonded Rotaxanes: Active Role of the Axle and the End Station. *J. Am. Chem. Soc.* **2019**, *141*, 19118–19129.
